# Periodic Trends in Low-Valent Group 14 and Group 15 Compounds: Theoretical Photoelectron Spectroscopy of Tetrylenes, Tetrylones, and Singlet Pnictinidenes

**DOI:** 10.1021/acsomega.6c04637

**Published:** 2026-07-29

**Authors:** Jeanet Conradie, Kristian Torstensen, Abhik Ghosh

**Affiliations:** † Department of Chemistry, University of Tromsø N-9037, Tromsø, Norway; ‡ Department of Chemistry, 37702 University of the Free State 9300, Bloemfontein, Republic of South Africa

## Abstract

Scalar-relativistic DFT calculations have been used to calculate the lowest ionization potentials (IPs) of six series of low-valent Group 14 (tetrel) and Group 15 (pnictogen) compounds with a view to uncovering periodic trends, especially the impact of scandide contraction and the inert pair effect. The compounds studied include: (a, b) EF_2_ and ECl_2_ (where E is a tetrel), (c) *N*,*N*′-dimethylimidazol-2-ylidine and its higher tetrel analogues (tetrylenes), (d, e) a carbodiphosphorane and a carbodicarbene and their higher tetrel analogues (tetrylones), and (f) a series of singlet pnictinidenes based on a pincer ligand. For the three series of tetrylenes studied (a–c), the IPs of the tetrel-based lone pairs exhibit a distinct crest at germanium, reflecting an electronic manifestation of scandide contraction. For the two tetrylone series and the singlet pnictinidenes (d–f), the IPs of the two lone pairs exhibit divergent trends. The lower of the two IPs, corresponding to ionization of the π-type lone pair is found to decrease monotonically down the groups. The higher IP, corresponding to ionization of the σ-type lone pair, in contrast, increases down the groups, reflecting the combined influence of scandide contraction and the inert pair effect.

## Graphical abstract

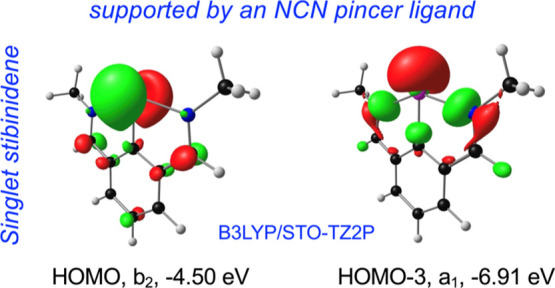

## Introduction

The chemistry of low-valent p-block element compounds is an exciting subfield of contemporary inorganic chemistry.^1–5^ Thus, monovalent Group 13 ^6–8^ (triel) and Group 15 ^9–13^ (pnictogen) compounds as well as zerovalent Group 14 (tetrel) compounds have been intensively studied in recent years.^14–18^ The latter compounds typically harbor two lone pairs on the tetrel atom, consistent with the notion that the latter has not used any of its valence electrons in bonding with other atoms (as a valence of zero implies^19^). A number of monovalent Group 15 compounds −singlet pnictinidenes^20–22^ −also share this feature, i.e., harbor two lone pairs on the pnictogen atom. Many pnictinidenes, on the other hand, are triplet species.^23,24^

Given the considerable insights that gas-phase photoelectron spectroscopy (PES) has yielded vis-à-vis the electronic structure of carbenes^25–27^ and other tetrylenes,^28,29^ we have long been interested in similar measurements on additional heavier tetrylenes as well as tetrylones and singlet pnictinidenes. That, unfortunately, is easier said than done. Gas-phase PES typically requires relatively large quantities of material (on the order of 100 mg) and specialized, custom-built equipment so the measurements are not readily applicable to exotic and sensitive compounds. A simple workaround, however, is available. For a wide variety of compounds, including carbenes,^30^ silylenes,^31,32^ strained hydrocarbons,^33^ fullerenes,^34–36^ porphyrins and related compounds,^37–41^ and metal–metal multiple-bonded complexes,^42,43^ DFT-derived ionization potentials (IPs) agree with gas-phase PES values to within a few tenths of an eV and often to within 0.1 eV. We accordingly undertook a DFT study of the lowest IPs of six series of low-valent Group 14 and 15 compounds (Scheme 1), encompassing tetrylenes, tetrylones, and singlet pnictinidenes, with a view to mapping out periodic trends. Following a similar study on low-valent Group 13 compounds,^44^ we were particularly interested in electronic manifestations of scandide contraction^45–47^ and of the inert pair effect.^48,49^

**Scheme 1:**
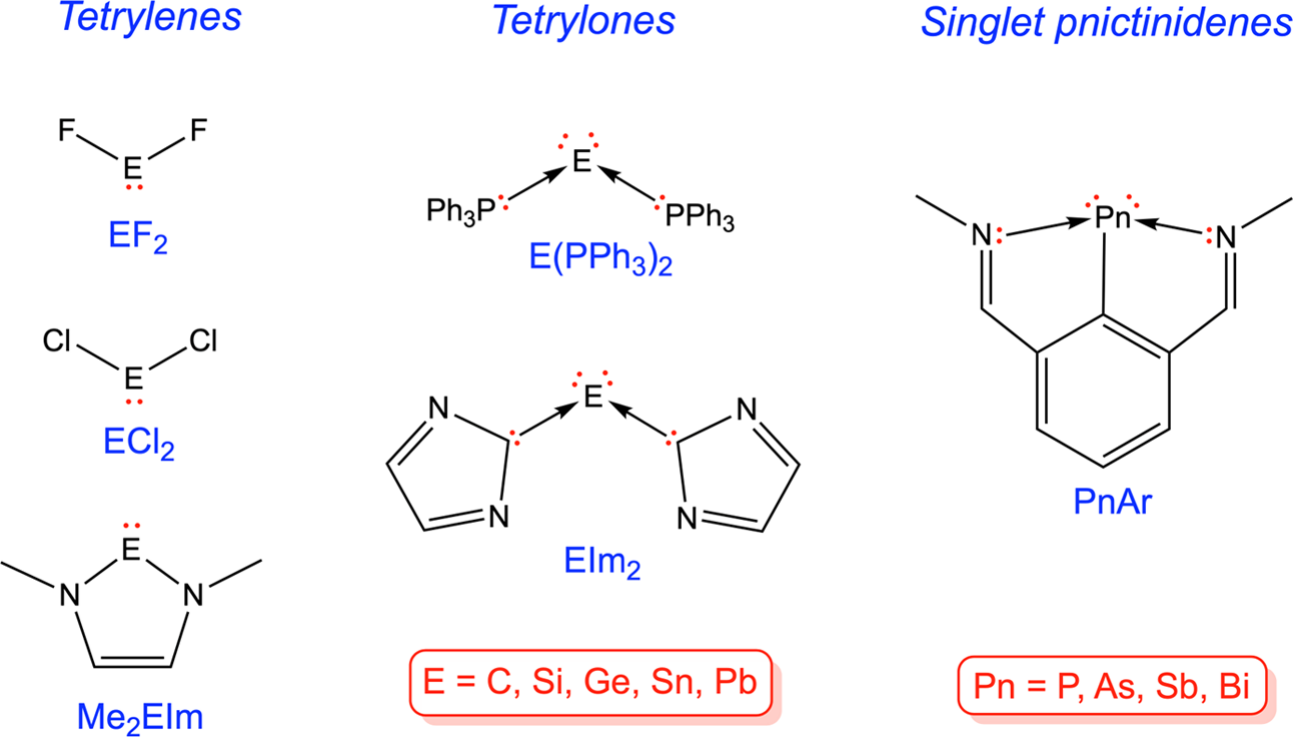
Low-Valent Tetrel and Pnictogen Compounds Studied in this Work

Scandide contraction refers to the unexpectedly small atomic and ionic radii of period-4 p-block elements as a result of poor screening of the nuclear charge by the 3d electrons.^45–47^ The phenomenon may be regarded as the main-group counterpart of lanthanide contraction. A well-known example is provided by gallium, which exhibits ionic and covalent radii very similar to those of aluminum.^50,51^ In a recent study,^44^ we found that the phenomenon manifests itself in the IPs of monovalent Group 13 compounds (trielylenes), with gallylenes exhibiting significantly higher IPs relative to aluminylenes. We wondered whether a similar effect might also be observed for Groups 14 and 15, with IPs cresting at germanium and arsenic.

Gas-phase IPs were calculated using a scalar-relativistic formalism, two different exchange–correlation functionals (OLYP^52,53^ and B3LYP,^54,55^ each augmented with D3(0)^56^ dispersion corrections) and all-electron ZORA-STO-TZ2P basis sets, as implemented in the ADF 2019 program system.^57^ (Given our focus on qualitative to semiquantitative insights, we have ignored spin–orbit effects in this study, which can be significant for period-6 p-block elements.^58–60^ ) The IPs were obtained via a ΔSCF protocol, i.e., as the difference in energy between the neutral and cationic states. The use of point group symmetry allowed the specification of the electron occupancy under each irrep, allowing the computation of multiple cationic states, and hence of multiple IPs, for each molecule studied. For vertical IPs, both the neutral and cationic states were assumed to be at the optimized neutral geometry. For adiabatic IPs, which are uncorrected for zero-point energies, the neutral and cationic states were separately optimized.

## Results and Discussion

Table 1 lists the calculated IPs of the different species studied and Figure 1 graphically depicts their periodic trends. Table 1 also includes nine relevant experimental IPs, obtained from gas-phase PES or other spectroscopic methods.^25–32^ Six of these values derive from gas-phase PES for *t*-butyl-substituted tetrylenes, whereas our calculations are based on the corresponding methyl analogues. Accordingly, a statistical analysis of computational errors does not appear to be justified in the present study. Nor do the data suggest a clear superiority for either of one functional over the other: both OLYP-D3 and B3LYP-D3 perform reasonably well, with errors <0.5 eV relatively to experimental values and in some cases as low as <0.1 eV. The OLYP-D3 values are in slightly better agreement with experimental values, compared with B3LYP-D3. Figure 2 depicts the HOMO and HOMO-1 of the different species studied. (Note: The discussion below focuses on vertical IPs, since gas-phase PES typically yields these quantities. The lowest adiabatic IP is also reported for each system; these may be useful for comparisons with trends in redox potentials, should they become available at some point.)

**Table 1:** DFT Calculations of Ionization Potentials (*I*, eV) for Selected Low-Valent Tetrel and Pnictogen Species^a^

		OLYP-D3				B3LYP-D3				Irrep		
molecule	PG	*I*_1v_	*I*_2v_	*I*_3v_	*I*_1a_	*I*_1v_	*I*_2v_	*I*_3v_	*I*_1a_	HOMO	HOMO–1	HOMO–2
CF_2_^25^	*C*_2v_	11.82 (12.26)	15.83	–	11.02 (11.36)	12.11 (12.26)	16.31	–	11.26 (11.36)	a_1_	b_1_	–
SiF_2_	*C*_2v_	10.71	14.37	–	10.35	11.10	14.86	–	10.70	a_1_	b_1_	–
GeF_2_	*C*_2v_	11.41	13.20	–	11.11	11.86	13.77	–	11.58	a_1_	b_1_	–
SnF_2_	*C*_2v_	10.77	12.34	–	10.58	11.26	12.92	–	11.06	a_1_	b_1_	–
PbF_2_	*C*_2v_	10.98	11.64	–	10.81	11.43	12.23	–	11.25	a_1_	b_1_	–
CCl_2_^26^	*C*_2v_	9.82	11.47	–	8.82 (9.27)	9.92	11.81	–	8.98 (9.27)	a_1_	b_1_	–
SiCl_2_^28^	*C*_2v_	9.71 (10.35)	11.01	–	9.22	9.95 (10.35)	11.33	–	9.48	a_1_	b_1_	–
GeCl_2_	*C*_2v_	10.01	10.65	–	9.74	10.33	10.97	–	10.06	a_1_	b_1_	–
SnCl_2_	*C*_2v_	9.75	10.27	–	9.54	10.08	10.66	–	9.88	a_1_	b_1_	–
PbCl_2_	*C*_2v_	9.81	9.97	–	9.44	10.15	10.34	–	9.73	a_1_	b_1_	–
Me_2_CIm^29^	*C*_2v_	7.87 (7.68)	8.29 (8.22)	–	7.46	7.99 (7.68)	8.42 (8.22)	–	8.10	a_1_	b_1_	–
Me_2_SiIm^29^	*C*_2v_	7.03 (6.96)	8.31 (8.01)	–	6.79	7.16 (6.96)	8.58 (8.01)	–	6.90	b_1_	a_1_	–
Me_2_GeIm^29^	*C*_2v_	6.63 (6.65)	8.74 (8.36)	–	6.39	6.79 (6.65)	9.09 (8.36)	–	6.54	b_1_	a_1_	–
Me_2_SnIm	*C*_2v_	6.14	8.61	–	5.95	6.33	8.93	–	6.13	b_1_	a_1_	–
Me_2_PbIm	*C*_2v_	5.80	8.28	8.93	5.63	5.99	8.57	9.26	5.81	b_1_	a_2_	a_1_
C(PPh_3_)_2_	*C*_2_	5.92	6.20	–	5.59	5.98	6.21	–	5.57	b	a	–
Si(PPh_3_)_2_	*C*_2_	4.79	6.63	–	4.57	4.80	6.83	–	4.60	b	a	–
Ge(PPh_3_)_2_	*C*_2_	4.33	6.70	–	4.34	4.63	7.13	–	4.44	b	a	–
Sn(PPh_3_)_2_	*C*_2_	4.21	6.72	–	4.11	4.39	7.13	–	4.26	b	a	–
Pb(PPh_3_)_2_	*C*_2_	4.01	6.91	–	3.90	4.25	7.35	–	4.13	b	a	–
CIm_2_	*C*_2_	5.46	6.09	–	5.30	5.55	6.12	–	5.39	b	a	–
siIm_2_	*C*_2_	4.73	6.65	–	4.51	4.72	6.88	–	4.52	b	a	–
GeIm_2_	*C*_2_	4.44	7.07	–	4.26	4.49	7.33	–	4.31	b	a	–
SnIm_2_	*C*_2_	4.09	7.03	–	3.97	4.17	7.33	–	4.07	b	a	–
PbIm_2_	*C*_2_	3.84	7.56	–	3.73	3.92	7.95	–	3.86	b	a	–
P-pincer	*C*_2v_	6.53	7.63	8.08	6.45	6.55	7.92	8.34	6.46	b_2_	a_1_	a_2_
As-pincer	*C*_2v_	6.27	8.01	8.02	6.19	6.35	8.35	8.30	6.27	b_2_	a_1_	a_2_
Sb-pincer	*C*_2v_	5.94	7.99	8.14	5.87	6.05	8.29	8.47	5.99	b_2_	a_2_	a_1_
Bipincer	*C*_2v_	5.65	8.01	8.27	5.65	5.86	8.22	8.81	5.80	b_2_	a_2_	a_1_

a The subscripts v and a refer to vertical and adiabatic IPs; PG = point group. The irrep (irreducible representation) refers to the occupied MO from which an electron is removed. Gas-phase experimental values (eV), when available from photoelectron spectroscopy,^25–29^ are indicated in parentheses.

**Figure 1:**
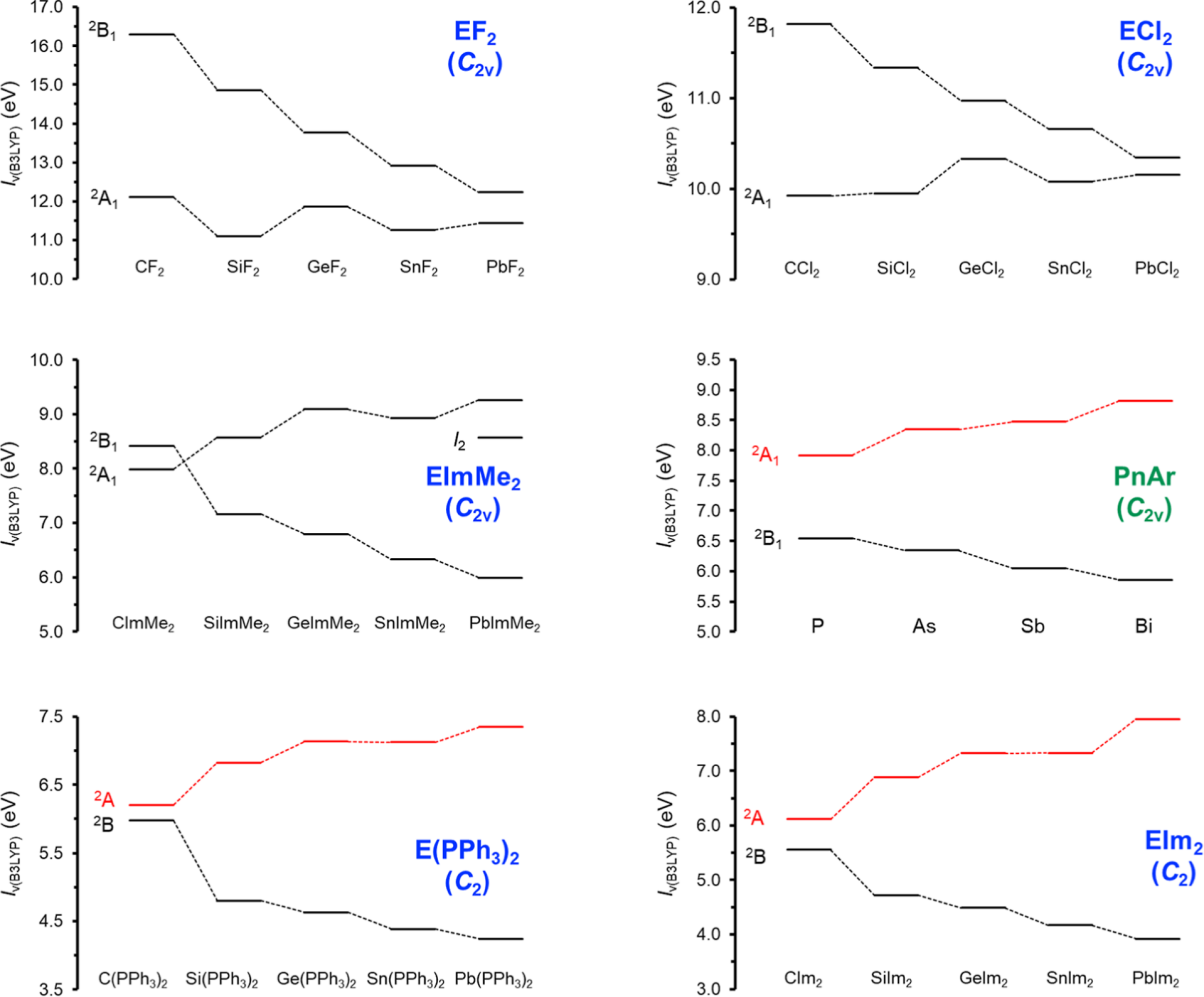
Periodic trends in vertical B3LYP-D3 IPs for the low-valent tetrel species studied. For molecules with two lone pairs on the atom of interest, the IP for the σ-type lone pair is indicated in red; state symmetries of the ionized states are indicated next to the vertical energy scales.

**Figure 2:**
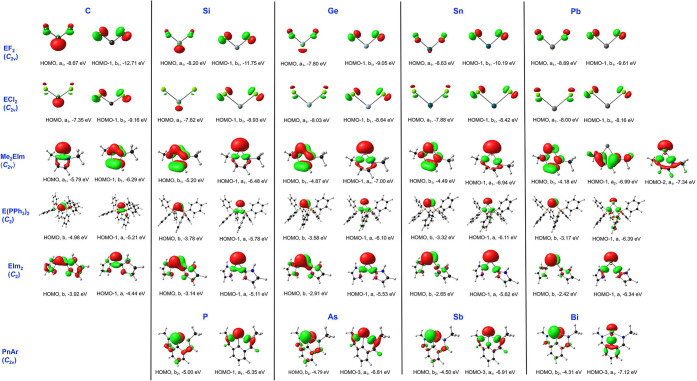
Selected B3LYP-D3/STO-TZ2P Frontier MOs for the molecules studied. Isodensity contours: 0.02 e/Å^–3^ for EF_2_ and ECl_2_ and 0.06 e/Å^–3^ for Me_2_EIm, E­(PPh_3_)_2_, EIm_2_, and PnAr.

For the carbenes CF_2_ and CCl_2_ and their higher heavier tetrylene analogues, the lowest IP corresponds to ionization from an a_1_-symmetry MO with partial tetrel lone pair character. The lone pair character of the MO, however, decreases for the heavier tetrels and is quite small for Sn and Pb. For both the EF_2_ and ECl_2_ series, the first IP exhibits a W-shaped or zigzag pattern characteristic of an effect known as “secondary periodicity”, which was identified by Biron over a century ago:^61–66^ the distinct crest at Ge appears to be indicative of scandide contraction, while the slightly elevated value for Pb, relative to Sn, appears to implicate a scalar relativistic effect.^67–70^ The small value of the latter effect reflects the low amplitude of the molecular orbital on the heavier tetrel atoms.

The second IP of the above EF_2_ and ECl_2_ series corresponds to ionization from a b_1_-symmetry MO with a nodal surface through the tetrel atom. Nevertheless, the IP decreases significantly down the group, consistent with decreasing electronegativity of the tetrel atom. In other words, the IPs of the halogen-based lone pairs are strongly modulated by their tetrel neighbors.

The *N*-heterocyclic tetrylenes Me_2_EIm exhibit an orbital reordering going down the group.^29,71,72^ Thus, the a_1_ MO with the highest amount of tetrel lone pair character corresponds to the HOMO for the carbene, but to the HOMO-1 for Si, Ge, and Sn, and the HOMO-2 for Pb (the HOMO in these cases being a π-type MO). As for the simple tetrylenes discussed above, the IP for this orbital exhibits a distinct crest at Ge, consistent with scandide contraction. Again, for this ionization, the IP for Pb is slightly higher than that for Sn, reflecting relativistic stabilization of the Pb lone pair;^67–70^ the effect, however, is small, because the MO in question has only partial tetrel lone pair character.

For the two tetrylone series studied, E­(PPh_3_)_2_ and EIm_2_, the IPs of the two tetrel-based lone pairs differ in their behavior going down the group. The IP for the π-type lone pair (corresponding to ionization of a b-symmetry MO) decreases down the group in a monotonic manner, a common periodic trend. On the other hand, the IP for the σ-type lone pair (corresponding to ionization of an a-symmetry MO) increases down the group, consistent with both scandide contraction and the inert pair effect (the latter also includes a relativistic effect).

For the singlet arylpnictinidenes studied, PnAr, the pincer ligand was slightly simplified relative to that experimentally used by Dostál and co-workers.^22^ Use of the simplified ligand was actually an important element in our computational strategy, which elevated the orbital energies of the two pnictogen lone pair-based MOs so they became the highest occupied MOs within their respective irreps. Group-theoretical manipulation of the orbital occupancies then allowed us to use standard ground-state DFT calculations to compute the IPs of the σ- and π-type lone pairs, leading to significant insights, as discussed below. (Note: For each molecule, the ordering of the three lowest IPs followed the orbital energy ordering of the three HOMOs. Orbital/IP reordering was observed as a function of position in the periodic table, but no discrepancy in orbital energy vs IP ordering was observed for any given compound.)

As for the tetrylones, the IPs of the two pnictogen-based lone pairs were found to vary differently going down the group. The lowest IP, corresponding to ionization of the π-type lone pair, was found to decrease monotonically down the group. In contrast, the second IP, corresponding to ionization of the σ-type lone pair, in contrast, was found to increase down the group, exhibiting significant jumps from P to As and from Sb to Bi. Again, this behavior is consistent with the combined influence of scandide contraction and the inert pair effect.

An interesting question concerns why the σ-type lone pairs of tetrylones and singlet pnictinidenes should be more susceptible to scandide contraction than the π-type lone pairs. While a detailed analysis has not been carried out at this point, it seems qualitatively reasonable that σ-type lone pairs, derived from s-orbitals, which lack angular nodes, should experience less screening of the nuclear charge than lone pairs based derived largely from p-orbitals, which do have angular nodes.

A few brief comments may be made on potential geometrical manifestations of scandide contraction. None seem obvious from Table 2, which lists selected optimized geometry parameters for the molecules studied. In other words, the element-substituent bond distance increases monotonically down both Groups 14 and 15. This scenario may be contrasted with that for Group 13. Indeed, Pyykkö has reported a slightly shorter single-bond covalent radius for gallium (124 pm) than for aluminum (126 pm).^50^ In our recent DFT study of low-valent Group 13 compounds (trielylenes), we also observed very similar, sometimes near-identical, single-bond distances involving aluminum and gallium.^44^ Table 2 does not reveal a similar trend for Si/Ge and P/As. For Groups 14 and 15, accordingly, scandide contraction appears to manifest itself primarily as an electronic effect, elevating ionization potentials by a few tenths of an eV, rather than as a geometrical effect.

**Table 2:** Selected Optimized Bond Distances (Å) and Bond Angles (°)^a^

	OLYP-D3				B3LYP-D3		
Molecule	PG	∠LEL (°)	d_E‑L_ (Å)		∠LEL (°)	d_E‑L_ (Å)	
CF_2_	*C*_2v_	105.5	1.322		104.8	1.305	
SiF_2_	*C*_2v_	102.7	1.624		101.2	1.612	
GeF_2_	*C*_2v_	97.9	1.776		97.9	1.761	
SnF_2_	*C*_2v_	96.4	1.963		96.3	1.954	
PbF_2_	*C*_2v_	96.5	2.069		96.5	2.058	
CCl_2_	*C*_2v_	108.2	1.744		109.7	1.733	
SiCl_2_	*C*_2v_	101.5	2.102		102.4	2.093	
GeCl_2_	*C*_2v_	101.2	2.207		101.5	2.209	
SnCl_2_	*C*_2v_	97.4	2.391		98.8	2.394	
PbCl_2_	*C*_2v_	101.2	2.498		99.5	2.488	
Me_2_CIm	*C*_2v_	101.4	1.368		102.2	1.360	
Me_2_SiIm	*C*_2v_	86.5	1.776		87.1	1.768	
Me_2_GeIm	*C*_2v_	83.0	1.898		83.2	1.894	
Me_2_SnIm	*C*_2v_	76.9	2.126		77.7	2.108	
Me_2_PbIm	*C*_2v_	74.5	2.232		75.0	2.222	
C(PPh_3_)_2_	*C*_2_	126.2	1.644		131.8	1.629	
Si(PPh_3_)_2_	*C*_2_	92.3	2.248		93.7	2.263	
Ge(PPh_3_)_2_	*C*_2_	93.3	2.342		92.0	2.376	
Sn(PPh_3_)_2_	*C*_2_	89.4	2.608		88.1	2.643	
Pb(PPh_3_)_2_	*C*_2_	89.9	2.726		87.1	2.756	
CIm_2_	*C*_2_	120.4	1.362		123.0	1.354	
SiIm_2_	*C*_2_	96.7	1.854		95.1	1.868	
GeIm_2_	*C*_2_	91.7	1.974		92.3	1.982	
SnIm_2_	*C*_2_	86.5	2.221		86.8	2.241	
PbIm_2_	*C*_2_	84.7	2.349		83.3	2.395	
		∠NPnC (°)	*d*_Pn‑C_ (Å)	*d*_Pn‑N_ (Å)	∠NPnC (°)	*d*_Pn‑C_ (Å)	*d*_Pn‑N_ (Å)
PAr	*C*_2v_	81.1	1.743	2.038	80.9	1.740	2.062
AsAr	*C*_2v_	78.2	1.861	2.190	77.9	1.863	2.209
SbAr	*C*_2v_	73.5	2.060	2.365	73.4	2.063	2.370
BiAr	*C*_2v_	71.5	2.152	2.474	71.3	2.155	2.479

a L = Ligand.

## Conclusion

DFT-based photoelectrons spectroscopy calculations on 6 series of low-valent tetrel and pnictogen compounds have yielded a wealth of insights into periodic trends in tetrylenes, tetrylones and singlet pnictinidenes:

- For the three tetrylene series studied, the IP of the tetrel-based lone pair exhibits a distinct crest for germylenes and a general zigzag patten down group, apparent electronic manifestations of scandide contraction and of the inert pair effect. The zigzag pattern is consistent with secondary periodicity, an effect identified by Biron over a century ago.
- For the two tetrylone series and for the singlet pnictinidene series, the calculated IPs for the two lone pairs of interest exhibit divergent trends. Thus, whereas the IP for the σ-type lone pair increases down the group, the IP for the π-type lone pair decreases, the latter being the more standard behavior expected on the basis of increasing atomic radii. The divergent behavior is plausibly ascribed to two effects. Scandide contraction is expected to affect valence s-orbitals (which lack angular nodes) much more strongly relative to p-orbitals. (Note that valence s orbitals contribute substantially only to σ-type lone pairs and not to π-type lone pairs.) A second factor is the inert pair effect, which is also specific to valence s-orbitals.
- Interestingly, unlike for Group 13, in which scandide contraction leads to near-identical ionic and single-bond covalent radii for Al and Ga, the bond distances involving Group 14 and 15 elements in a given class of compounds increase monotonically down the group in question. Thus, for Groups 14 and 15, scandide contraction appears to manifest itself primarily as an electronic, as opposed to a structural, effect.

While we view our results as well-calibrated against photoelectron spectra of selected tetrylenes and hence largely trustworthy, we look forward to eventual experimental confirmation of some of more interesting predicted results, especially those involving tetrylones and singlet pnictinidenes. We also look forward to connecting the present results to aspects of bonding and reactivity with transition metal centers, a subject of broad interest in the inorganic chemistry community and beyond.^73–75^

## Computational Methods

All DFT calculations employed the Zeroth Order Regular Approximation (ZORA) to the Dirac equation,^76^ either the OLYP^52^ or the B3LYP^53–55^ exchange–correlation functional, Grimme’s D3(0)^56^ dispersion correction, and all-electron ZORA-STO-TZ2P relativistic basis sets, all implemented in the ADF 2019 program system.^56,57^ Carefully chosen, fine integration grids and tight criteria for the SCF cycles and geometry optimization were employed; as these parameters are software-specific, readers aiming to reproduce the current results should test these parameters vis-à-vis the stability of all results, especially the optimized geometries. A sample input file is included in the Supporting Information for convenience. Point group symmetry was exploited, as indicated in Tables 1 and 2.

## Supplementary Material



## Notes

Optimized OLYP-D3 and B3LYP-D3 coordinates (PDF)
