# Supplementary material for: Periodic Trends in Low-Valent Group 14 and Group 15 Compounds: Theoretical Photoelectron Spectroscopy of Tetrylenes, Tetrylones, and Singlet Pnictinidenes
Source: ACS Omega. 2026 Jul 29;11(31):46746–53. doi: 10.1021/acsomega.6c04637 (PMC13470715; doi:10.1021/acsomega.6c04637)
Supplement: Supplementary file 1 [file ao6c04637_si_001.pdf]

Periodic Trends in Low-Valent Group 14 and Group 15 Compounds: Theoretical Photoelectron Spectroscopy of Tetrylenes, Tetrylones, and Singlet Pnictinidenes

Jeanet Conradie[a,b] Kristian Torstensen[a] and Abhik Ghosh*[,a]

[a]Department of Chemistry, University of Tromsø, N-9037 Tromsø, Norway;

[b]Department of Chemistry, University of the Free State, 9300 Bloemfontein, Republic of South Africa.

Supporting information

Table of Contents

Example input file for ADF 3

Optimized coordinates 4

1. CF_2, C_{2v} 4
2. SiF_2, C_{2v} 4
3. GeF_2, C_{2v} 4
4. SnF_2, C_{2v} 5
5. PbF_2, C_{2v} 5
6. CCl_2, C_{2v} 6
7. $SiCl_2$, C_{2v} 6
8. $GeCl_2$, C_{2v} 7
9. $SnCl_2$, C_{2v} 7
10. $PbCl_2$, C_{2v} 7
11. Me_2CIm, C_{2v} 8
12. Me_2SiIm, C_{2v} 9
13. Me_2GeIm, C_{2v} 11
14. Me_2SnIm, C_{2v} 12
15. Me_2PbIm, C_{2v} 13
16. $C(PPh_3)_2$, C_2 15
17. $Si(PPh_3)_2$, C_2 20

18. $Ge(PPh_3)_2$, C_2 26
19. $Sn(PPh_3)_2$, C_2 32
20. $Pb(PPh_3)_2$, C_2 37
21. CIm_2, C_2 43
22. $SiIm_2$, C_2 44
23. $GeIm_2$, C_2 46
24. $SnIm_2$, C_2 48
25. $PbIm_2$, C_2 49
26. PAr, C_{2v} 51
27. AsAr, C_{2v} 53
28. SbAr, C_{2v} 55
29. BiAr, C_{2v} 57

Example input file for ADF

```
Title GeCl2 neutral

UNITS
  length angstrom
END

Atoms Cartesian
Ge       0.000000000      0.000000000     -0.910319772
Cl       1.412603531      0.000000000      0.156193831
Cl      -1.412603531      0.000000000      0.156193831
end

SYMMETRY C(2v)

CHARGE 0

INTEGRATION
  accint 6.0
End

RELATIVISTIC Scalar ZORA

GEOMETRY
  Iterations 500
  Converge e=1.0E-3 grad=1.0E-3 rad=1.0E-2
End

SCF
Iterations 500
Converge 1.0E-7
END

BASIS
  Type ZORA/TZ2P
  Core none
END

XC
  DISPERSION Grimme3
  hybrid B3LYP
END
endinput
```

Optimized coordinates

1. CF_2, C_{2v}

OLYP, NEUTRAL

C	0.000000000	0.000000000	-0.732327000
F	1.052307000	0.000000000	0.067197000
F	-1.052307000	0.000000000	0.067197000

B3LYP, NEUTRAL

C	0.000000000	0.000000000	-0.730342000
F	1.034163000	0.000000000	0.066205000
F	-1.034163000	0.000000000	0.066205000

OLYP, GROUND-STATE CATION

C	0.000000000	0.000000000	-0.579517000
F	1.087126000	0.000000000	-0.009208000
F	-1.087126000	0.000000000	-0.009208000

B3LYP, GROUND-STATE CATION

C	0.000000000	0.000000000	-0.576130000
F	1.083866000	0.000000000	-0.010901000
F	-1.083866000	0.000000000	-0.010901000

2. SiF_2, C_{2v}

OLYP, NEUTRAL

Si	0.000000000	0.000000000	-0.875650000
F	1.267672000	0.000000000	0.138859000
F	-1.267672000	0.000000000	0.138859000

B3LYP, NEUTRAL

Si	0.000000000	0.000000000	-0.881323000
F	1.245783000	0.000000000	0.141695000
F	-1.245783000	0.000000000	0.141695000

OLYP, GROUND-STATE CATION

Si	0.000000000	0.000000000	-0.698532000
F	1.358832000	0.000000000	0.050300000
F	-1.358832000	0.000000000	0.050300000

B3LYP, GROUND-STATE CATION

Si	0.000000000	0.000000000	-0.711770000
F	1.336255000	0.000000000	0.056919000
F	-1.336255000	0.000000000	0.056919000

3. GeF_2, C_{2v}

OLYP, NEUTRAL

Ge	0.000000000	0.000000000	-0.976724000
F	1.339383000	0.000000000	0.189396000
F	-1.339383000	0.000000000	0.189396000

B3LYP, NEUTRAL

Ge	0.000000000	0.000000000	-0.969922000
F	1.328176000	0.000000000	0.185995000
F	-1.328176000	0.000000000	0.185995000

OLYP, GROUND-STATE CATION

Ge	0.000000000	0.000000000	-0.796537000
F	1.448794000	0.000000000	0.099303000
F	-1.448794000	0.000000000	0.099303000

B3LYP, GROUND-STATE CATION

Ge	0.000000000	0.000000000	-0.789368000
F	1.445708000	0.000000000	0.095718000
F	-1.445708000	0.000000000	0.095718000

4. SnF_2, C_{2v}

OLYP, NEUTRAL

Sn	0.000000000	0.000000000	-1.071387000
F	1.463165000	0.000000000	0.236727000
F	-1.463165000	0.000000000	0.236727000

B3LYP, NEUTRAL

Sn	0.000000000	0.000000000	-1.068291000
F	1.455386000	0.000000000	0.235179000
F	-1.455386000	0.000000000	0.235179000

OLYP, GROUND-STATE CATION

Sn	0.000000000	0.000000000	-0.897209000
F	1.589427000	0.000000000	0.149639000
F	-1.589427000	0.000000000	0.149639000

B3LYP, GROUND-STATE CATION

Sn	0.000000000	0.000000000	-0.858631000
F	1.621650000	0.000000000	0.130349000
F	-1.621650000	0.000000000	0.130349000

5. PbF_2, C_{2v}

OLYP, NEUTRAL

Pb	0.000000000	0.000000000	-1.118031000
F	1.543891000	0.000000000	0.260049000
F	-1.543891000	0.000000000	0.260049000

B3LYP, NEUTRAL

Pb	0.000000000	0.000000000	-1.112934000
F	1.534881000	0.000000000	0.257501000
F	-1.534881000	0.000000000	0.257501000

OLYP, GROUND-STATE CATION

Pb	0.000000000	0.000000000	-0.807615000
F	1.826911000	0.000000000	0.104841000
F	-1.826911000	0.000000000	0.104841000

B3LYP, GROUND-STATE CATION

Pb	0.000000000	0.000000000	-0.743006000
F	1.870916000	0.000000000	0.072537000
F	-1.870916000	0.000000000	0.072537000

6. CCl_2, C_{2v}

OLYP, NEUTRAL

C	0.000000000	0.000000000	-0.881568000
Cl	1.412683000	0.000000000	0.141818000
Cl	-1.412683000	0.000000000	0.141818000

B3LYP, NEUTRAL

C	0.000000000	0.000000000	-0.864581000
Cl	1.416686000	0.000000000	0.133324000
Cl	-1.416686000	0.000000000	0.133324000

OLYP, GROUND-STATE CATION

C	0.000000000	0.000000000	-0.587033000
Cl	1.489038000	0.000000000	-0.005449000
Cl	-1.489038000	0.000000000	-0.005449000

B3LYP, GROUND-STATE CATION

C	0.000000000	0.000000000	-0.598537000
Cl	1.478278000	0.000000000	0.000303000
Cl	-1.478278000	0.000000000	0.000303000

7. $SiCl_2$, C_{2v}

OLYP, NEUTRAL

Si	0.000000000	0.000000000	-1.086154000
Cl	1.627362000	0.000000000	0.244111000
Cl	-1.627362000	0.000000000	0.244111000

B3LYP, NEUTRAL

Si	0.000000000	0.000000000	-1.074147000
Cl	1.631150000	0.000000000	0.238108000
Cl	-1.631150000	0.000000000	0.238108000

OLYP, GROUND-STATE CATION

Si	0.000000000	0.000000000	-0.838415000
Cl	1.736208000	0.000000000	0.120241000
Cl	-1.736208000	0.000000000	0.120241000

B3LYP, GROUND-STATE CATION

Si	0.000000000	0.000000000	-0.835275000

Cl	1.728721000	0.000000000	0.118671000
Cl	-1.728721000	0.000000000	0.118671000

8. $GeCl_2$, C_{2v}

OLYP, NEUTRAL

Ge	0.000000000	0.000000000	-1.132898000
Cl	1.706107000	0.000000000	0.267483000
Cl	-1.706107000	0.000000000	0.267483000

B3LYP, NEUTRAL

Ge	0.000000000	0.000000000	-1.131076000
Cl	1.709985000	0.000000000	0.266572000
Cl	-1.709985000	0.000000000	0.266572000

OLYP, GROUND-STATE CATION

Ge	0.000000000	0.000000000	-0.911933000
Cl	1.814819000	0.000000000	0.157000000
Cl	-1.814819000	0.000000000	0.157000000

B3LYP, GROUND-STATE CATION

Ge	0.000000000	0.000000000	-0.902242000
Cl	1.820402000	0.000000000	0.152155000
Cl	-1.820402000	0.000000000	0.152155000

9. $SnCl_2$, C_{2v}

OLYP, NEUTRAL

Sn	0.000000000	0.000000000	-1.251449000
Cl	1.796137000	0.000000000	0.326758000
Cl	-1.796137000	0.000000000	0.326758000

B3LYP, NEUTRAL

Sn	0.000000000	0.000000000	-1.237570000
Cl	1.817751000	0.000000000	0.319819000
Cl	-1.817751000	0.000000000	0.319819000

OLYP, GROUND-STATE CATION

Sn	0.000000000	0.000000000	-1.035770000
Cl	1.926678000	0.000000000	0.218919000
Cl	-1.926678000	0.000000000	0.218919000

B3LYP, GROUND-STATE CATION

Sn	0.000000000	0.000000000	-0.988254000
Cl	1.981910000	0.000000000	0.195161000
Cl	-1.981910000	0.000000000	0.195161000

10. $PbCl_2$, C_{2v}

OLYP, NEUTRAL

Pb	0.000000000	0.000000000	-1.256183000

Cl	1.929956000	0.000000000	0.329125000
Cl	-1.929956000	0.000000000	0.329125000

B3LYP, NEUTRAL

Pb	0.000000000	0.000000000	-1.270509000
Cl	1.898944000	0.000000000	0.336289000
Cl	-1.898944000	0.000000000	0.336289000

OLYP, GROUND-STATE CATION

Pb	0.000000000	0.000000000	-1.070073000
Cl	2.070234000	0.000000000	0.236070000
Cl	-2.070234000	0.000000000	0.236070000

B3LYP, GROUND-STATE CATION

Pb	0.000000000	0.000000000	-0.995608000
Cl	2.133348000	0.000000000	0.198838000
Cl	-2.133348000	0.000000000	0.198838000

11. Me_2CIm, C_{2v}

OLYP, NEUTRAL

C	0.000000000	0.000000000	0.992040000
C	0.000000000	0.679877000	-1.211106000
C	0.000000000	2.442464000	0.576199000
C	0.000000000	-0.679877000	-1.211106000
C	0.000000000	-2.442464000	0.576199000
H	0.000000000	1.385781000	-2.028576000
H	0.000000000	3.104512000	-0.293972000
H	0.000000000	-1.385781000	-2.028576000
H	0.000000000	-3.104512000	-0.293972000
H	0.890359000	2.637149000	1.181364000
H	0.890359000	-2.637149000	1.181364000
H	-0.890359000	2.637149000	1.181364000
H	-0.890359000	-2.637149000	1.181364000
N	0.000000000	1.058441000	0.124989000
N	0.000000000	-1.058441000	0.124989000

B3LYP, NEUTRAL

C	0.000000000	0.000000000	0.973264000
C	0.000000000	0.675707000	-1.213131000
C	0.000000000	2.436937000	0.578755000
C	0.000000000	-0.675707000	-1.213131000
C	0.000000000	-2.436937000	0.578755000
H	0.000000000	1.375032000	-2.029545000
H	0.000000000	3.105820000	-0.279803000
H	0.000000000	-1.375032000	-2.029545000
H	0.000000000	-3.105820000	-0.279803000
H	0.884754000	2.630862000	1.182119000
H	0.884754000	-2.630862000	1.182119000
H	-0.884754000	2.630862000	1.182119000
H	-0.884754000	-2.630862000	1.182119000

N	0.000000000	1.058335000	0.119135000
N	0.000000000	-1.058335000	0.119135000

OLYP, GROUND-STATE CATION

C	0.000000000	0.000000000	0.851066000
C	0.000000000	0.682413000	-1.215965000
C	0.000000000	2.487469000	0.600212000
C	0.000000000	-0.682413000	-1.215965000
C	0.000000000	-2.487469000	0.600212000
H	0.000000000	1.394425000	-2.027742000
H	0.000000000	3.147775000	-0.267342000
H	0.000000000	-1.394425000	-2.027742000
H	0.000000000	-3.147775000	-0.267342000
H	0.897392000	2.658527000	1.199888000
H	0.897392000	-2.658527000	1.199888000
H	-0.897392000	2.658527000	1.199888000
H	-0.897392000	-2.658527000	1.199888000
N	0.000000000	1.099562000	0.111808000
N	0.000000000	-1.099562000	0.111808000

B3LYP, GROUND-STATE CATION

C	0.000000000	0.000000000	0.961912000
C	0.000000000	0.713098000	-1.197750000
C	0.000000000	2.466944000	0.580029000
C	0.000000000	-0.713098000	-1.197750000
C	0.000000000	-2.466944000	0.580029000
H	0.000000000	1.386474000	-2.040925000
H	0.000000000	3.159107000	-0.256532000
H	0.000000000	-1.386474000	-2.040925000
H	0.000000000	-3.159107000	-0.256532000
H	0.886368000	2.611594000	1.193124000
H	0.886368000	-2.611594000	1.193124000
H	-0.886368000	2.611594000	1.193124000
H	-0.886368000	-2.611594000	1.193124000
N	0.000000000	1.088817000	0.074255000
N	0.000000000	-1.088817000	0.074255000

12. Me_2SiIm, C_{2v}

OLYP, NEUTRAL

C	0.000000000	0.681178000	-1.012337000
N	0.000000000	-1.217585000	0.263166000
C	0.000000000	-0.681178000	-1.012337000
N	0.000000000	1.217585000	0.263166000
H	0.000000000	-1.319994000	-1.887913000
H	0.000000000	1.319994000	-1.887913000
C	0.000000000	2.669791000	0.439795000
H	-0.889575000	2.998100000	0.990991000
H	0.889575000	2.998100000	0.990991000
H	0.000000000	3.164753000	-0.536665000
C	0.000000000	-2.669791000	0.439795000

H	0.000000000	-3.164753000	-0.536665000
H	0.889575000	-2.998100000	0.990991000
H	-0.889575000	-2.998100000	0.990991000
Si	0.000000000	0.000000000	1.556509000

B3LYP, NEUTRAL

C	0.000000000	0.675927000	-1.008965000
N	0.000000000	-1.217832000	0.262823000
C	0.000000000	-0.675927000	-1.008965000
N	0.000000000	1.217832000	0.262823000
H	0.000000000	-1.308174000	-1.883639000
H	0.000000000	1.308174000	-1.883639000
C	0.000000000	2.667732000	0.440354000
H	-0.884475000	2.997070000	0.986569000
H	0.884475000	2.997070000	0.986569000
H	0.000000000	3.163985000	-0.529474000
C	0.000000000	-2.667732000	0.440354000
H	0.000000000	-3.163985000	-0.529474000
H	0.884475000	-2.997070000	0.986569000
H	-0.884475000	-2.997070000	0.986569000
Si	0.000000000	0.000000000	1.544085000

OLYP, GROUND-STATE CATION

C	0.000000000	0.709918000	-1.001909000
N	0.000000000	-1.243704000	0.215759000
C	0.000000000	-0.709918000	-1.001909000
N	0.000000000	1.243704000	0.215759000
H	0.000000000	-1.317913000	-1.902337000
H	0.000000000	1.317913000	-1.902337000
C	0.000000000	2.697911000	0.435418000
H	-0.889503000	2.980755000	1.008608000
H	0.889503000	2.980755000	1.008608000
H	0.000000000	3.223489000	-0.521983000
C	0.000000000	-2.697911000	0.435418000
H	0.000000000	-3.223489000	-0.521983000
H	0.889503000	-2.980755000	1.008608000
H	-0.889503000	-2.980755000	1.008608000
Si	0.000000000	0.000000000	1.568234000

B3LYP, GROUND-STATE CATION

C	0.000000000	0.706470000	-0.991634000
N	0.000000000	-1.238859000	0.220955000
C	0.000000000	-0.706470000	-0.991634000
N	0.000000000	1.238859000	0.220955000
H	0.000000000	-1.308313000	-1.890392000
H	0.000000000	1.308313000	-1.890392000
C	0.000000000	2.695380000	0.432884000
H	-0.885560000	2.985423000	0.995881000
H	0.885560000	2.985423000	0.995881000
H	0.000000000	3.213455000	-0.522924000
C	0.000000000	-2.695380000	0.432884000

H	0.000000000	-3.213455000	-0.522924000
H	0.885560000	-2.985423000	0.995881000
H	-0.885560000	-2.985423000	0.995881000
Si	0.000000000	0.000000000	1.571260000

13.Me_2GeIm, C_{2v}

OLYP, NEUTRAL

C	0.000000000	0.683945000	-0.973600000
N	0.000000000	-1.257647000	0.274545000
C	0.000000000	-0.683945000	-0.973600000
N	0.000000000	1.257647000	0.274545000
H	0.000000000	-1.300954000	-1.865861000
H	0.000000000	1.300954000	-1.865861000
C	0.000000000	2.710871000	0.410902000
H	-0.888882000	3.055316000	0.954182000
H	0.888882000	3.055316000	0.954182000
H	0.000000000	3.185413000	-0.576377000
C	0.000000000	-2.710871000	0.410902000
H	0.000000000	-3.185413000	-0.576377000
H	0.888882000	-3.055316000	0.954182000
H	-0.888882000	-3.055316000	0.954182000
Ge	0.000000000	0.000000000	1.696619000

B3LYP, NEUTRAL

C	0.000000000	0.679053000	-0.965150000
N	0.000000000	-1.257936000	0.277758000
C	0.000000000	-0.679053000	-0.965150000
N	0.000000000	1.257936000	0.277758000
H	0.000000000	-1.287590000	-1.858046000
H	0.000000000	1.287590000	-1.858046000
C	0.000000000	2.709629000	0.409136000
H	-0.883988000	3.058217000	0.945334000
H	0.883988000	3.058217000	0.945334000
H	0.000000000	3.180238000	-0.574788000
C	0.000000000	-2.709629000	0.409136000
H	0.000000000	-3.180238000	-0.574788000
H	0.883988000	-3.058217000	0.945334000
H	-0.883988000	-3.058217000	0.945334000
Ge	0.000000000	0.000000000	1.693409000

OLYP, GROUND-STATE CATION

C	0.000000000	0.710024000	-0.922212000
N	0.000000000	-1.288492000	0.265770000
C	0.000000000	-0.710024000	-0.922212000
N	0.000000000	1.288492000	0.265770000
H	0.000000000	-1.290908000	-1.842771000
H	0.000000000	1.290908000	-1.842771000
C	0.000000000	2.750664000	0.381968000
H	-0.890429000	3.077599000	0.929333000
H	0.890429000	3.077599000	0.929333000

H	0.000000000	3.208935000	-0.611460000
C	0.000000000	-2.750664000	0.381968000
H	0.000000000	-3.208935000	-0.611460000
H	0.890429000	-3.077599000	0.929333000
H	-0.890429000	-3.077599000	0.929333000
Ge	0.000000000	0.000000000	1.792639000

B3LYP, GROUND-STATE CATION

C	0.000000000	0.708253000	-0.937139000
N	0.000000000	-1.283422000	0.246807000
C	0.000000000	-0.708253000	-0.937139000
N	0.000000000	1.283422000	0.246807000
H	0.000000000	-1.287690000	-1.852200000
H	0.000000000	1.287690000	-1.852200000
C	0.000000000	2.743627000	0.393016000
H	-0.885054000	3.058806000	0.943723000
H	0.885054000	3.058806000	0.943723000
H	0.000000000	3.222915000	-0.583185000
C	0.000000000	-2.743627000	0.393016000
H	0.000000000	-3.222915000	-0.583185000
H	0.885054000	-3.058806000	0.943723000
H	-0.885054000	-3.058806000	0.943723000
Ge	0.000000000	0.000000000	1.743072000

14. Me_2SnIm**,** C_{2v}

OLYP, NEUTRAL

C	0.000000000	0.686761000	-0.876178000
N	0.000000000	-1.322784000	0.334626000
C	0.000000000	-0.686761000	-0.876178000
N	0.000000000	1.322784000	0.334626000
H	0.000000000	-1.268022000	-1.795270000
H	0.000000000	1.268022000	-1.795270000
C	0.000000000	2.782378000	0.342327000
H	-0.889321000	3.176271000	0.851870000
H	0.889321000	3.176271000	0.851870000
H	0.000000000	3.174353000	-0.682540000
C	0.000000000	-2.782378000	0.342327000
H	0.000000000	-3.174353000	-0.682540000
H	0.889321000	-3.176271000	0.851870000
H	-0.889321000	-3.176271000	0.851870000
Sn	0.000000000	0.000000000	1.999153000

B3LYP, NEUTRAL

C	0.000000000	0.682538000	-0.882108000
N	0.000000000	-1.322438000	0.323020000
C	0.000000000	-0.682538000	-0.882108000
N	0.000000000	1.322438000	0.323020000
H	0.000000000	-1.257082000	-1.799508000
H	0.000000000	1.257082000	-1.799508000
C	0.000000000	2.780103000	0.348192000

H	-0.883051000	3.167611000	0.859884000
H	0.883051000	3.167611000	0.859884000
H	0.000000000	3.186156000	-0.665152000
C	0.000000000	-2.780103000	0.348192000
H	0.000000000	-3.186156000	-0.665152000
H	0.883051000	-3.167611000	0.859884000
H	-0.883051000	-3.167611000	0.859884000
Sn	0.000000000	0.000000000	1.964138000

OLYP, GROUND-STATE CATION

C	0.000000000	0.711647000	-0.866700000
N	0.000000000	-1.337407000	0.294971000
C	0.000000000	-0.711647000	-0.866700000
N	0.000000000	1.337407000	0.294971000
H	0.000000000	-1.266354000	-1.804695000
H	0.000000000	1.266354000	-1.804695000
C	0.000000000	2.799698000	0.331564000
H	-0.889077000	3.158518000	0.862980000
H	0.889077000	3.158518000	0.862980000
H	0.000000000	3.215780000	-0.681089000
C	0.000000000	-2.799698000	0.331564000
H	0.000000000	-3.215780000	-0.681089000
H	0.889077000	-3.158518000	0.862980000
H	-0.889077000	-3.158518000	0.862980000
Sn	0.000000000	0.000000000	2.052542000

B3LYP, GROUND-STATE CATION

C	0.000000000	0.709480000	-0.866044000
N	0.000000000	-1.338775000	0.287346000
C	0.000000000	-0.709480000	-0.866044000
N	0.000000000	1.338775000	0.287346000
H	0.000000000	-1.256407000	-1.802350000
H	0.000000000	1.256407000	-1.802350000
C	0.000000000	2.804927000	0.336004000
H	-0.884005000	3.158623000	0.865735000
H	0.884005000	3.158623000	0.865735000
H	0.000000000	3.227952000	-0.667540000
C	0.000000000	-2.804927000	0.336004000
H	0.000000000	-3.227952000	-0.667540000
H	0.884005000	-3.158623000	0.865735000
H	-0.884005000	-3.158623000	0.865735000
Sn	0.000000000	0.000000000	2.014794000

15. Me_2PbIm, C_{2v}

OLYP, NEUTRAL

C	0.000000000	0.689413000	-0.843975000
N	0.000000000	-1.350909000	0.344895000
C	0.000000000	-0.689413000	-0.843975000
N	0.000000000	1.350909000	0.344895000
H	0.000000000	-1.251912000	-1.777114000

H	0.000000000	1.251912000	-1.777114000
C	0.000000000	2.807573000	0.317849000
H	-0.888858000	3.214353000	0.819502000
H	0.888858000	3.214353000	0.819502000
H	0.000000000	3.180945000	-0.715437000
C	0.000000000	-2.807573000	0.317849000
H	0.000000000	-3.180945000	-0.715437000
H	0.888858000	-3.214353000	0.819502000
H	-0.888858000	-3.214353000	0.819502000
Pb	0.000000000	0.000000000	2.122119000

B3LYP, NEUTRAL

C	0.000000000	0.685457000	-0.840880000
N	0.000000000	-1.352248000	0.340036000
C	0.000000000	-0.685457000	-0.840880000
N	0.000000000	1.352248000	0.340036000
H	0.000000000	-1.240246000	-1.772434000
H	0.000000000	1.240246000	-1.772434000
C	0.000000000	2.808370000	0.318390000
H	-0.883040000	3.211681000	0.818501000
H	0.883040000	3.211681000	0.818501000
H	0.000000000	3.186098000	-0.707163000
C	0.000000000	-2.808370000	0.318390000
H	0.000000000	-3.186098000	-0.707163000
H	0.883040000	-3.211681000	0.818501000
H	-0.883040000	-3.211681000	0.818501000
Pb	0.000000000	0.000000000	2.102663000

OLYP, GROUND-STATE CATION

C	0.000000000	0.713620000	-0.825030000
N	0.000000000	-1.373121000	0.313267000
C	0.000000000	-0.713620000	-0.825030000
N	0.000000000	1.373121000	0.313267000
H	0.000000000	-1.245699000	-1.777619000
H	0.000000000	1.245699000	-1.777619000
C	0.000000000	2.836301000	0.302351000
H	-0.888862000	3.211815000	0.822895000
H	0.888862000	3.211815000	0.822895000
H	0.000000000	3.223145000	-0.722602000
C	0.000000000	-2.836301000	0.302351000
H	0.000000000	-3.223145000	-0.722602000
H	0.888862000	-3.211815000	0.822895000
H	-0.888862000	-3.211815000	0.822895000
Pb	0.000000000	0.000000000	2.180251000

B3LYP, GROUND-STATE CATION

C	0.000000000	0.712442000	-0.824439000
N	0.000000000	-1.368623000	0.306960000
C	0.000000000	-0.712442000	-0.824439000
N	0.000000000	1.368623000	0.306960000
H	0.000000000	-1.239858000	-1.773436000

H	0.000000000	1.239858000	-1.773436000
C	0.000000000	2.833104000	0.304174000
H	-0.883574000	3.204538000	0.822581000
H	0.883574000	3.204538000	0.822581000
H	0.000000000	3.225284000	-0.712685000
C	0.000000000	-2.833104000	0.304174000
H	0.000000000	-3.225284000	-0.712685000
H	0.883574000	-3.204538000	0.822581000
H	-0.883574000	-3.204538000	0.822581000
Pb	0.000000000	0.000000000	2.161091000

16.C(PPh$_3$)$_2$, *C$_2$*

OLYP, NEUTRAL

C	0.000000000	0.000000000	1.126551000
C	0.024418000	2.316875000	-0.744973000
C	0.239495000	1.964150000	-2.083815000
C	0.680042000	3.441687000	-0.225655000
C	1.083484000	2.723985000	-2.889851000
C	1.344330000	-2.041394000	2.986228000
C	1.528163000	4.199591000	-1.031095000
C	1.584814000	-2.304047000	1.634715000
C	1.731512000	3.844238000	-2.365988000
C	1.883097000	-2.874377000	3.967819000
C	2.324886000	-0.623799000	-0.591574000
C	2.366393000	-3.406516000	1.270094000
C	2.661092000	-3.974502000	3.602238000
C	2.857228000	0.627592000	-0.258711000
C	2.901270000	-4.240190000	2.251316000
C	2.910296000	-1.366887000	-1.623725000
C	3.952259000	1.134784000	-0.953113000
C	4.006363000	-0.856970000	-2.320825000
C	4.528812000	0.394888000	-1.987421000
C	-0.024418000	-2.316875000	-0.744973000
C	-0.239495000	-1.964150000	-2.083815000
C	-0.680042000	-3.441687000	-0.225655000
C	-1.083484000	-2.723985000	-2.889851000
C	-1.344330000	2.041394000	2.986228000
C	-1.528163000	-4.199591000	-1.031095000
C	-1.584814000	2.304047000	1.634715000
C	-1.731512000	-3.844238000	-2.365988000
C	-1.883097000	2.874377000	3.967819000
C	-2.324886000	0.623799000	-0.591574000
C	-2.366393000	3.406516000	1.270094000
C	-2.661092000	3.974502000	3.602238000
C	-2.857228000	-0.627592000	-0.258711000
C	-2.901270000	4.240190000	2.251316000
C	-2.910296000	1.366887000	-1.623725000
C	-3.952259000	-1.134784000	-0.953113000
C	-4.006363000	0.856970000	-2.320825000
C	-4.528812000	-0.394888000	-1.987421000

H	0.261259000	-1.094110000	-2.494183000
H	0.526010000	3.720642000	0.812744000
H	0.733667000	-1.178183000	3.239119000
H	1.242862000	2.437797000	-3.927174000
H	1.695679000	-2.664756000	5.019073000
H	2.029766000	5.071592000	-0.616504000
H	2.380727000	1.200622000	0.529862000
H	2.395062000	4.434488000	-2.993637000
H	2.499642000	-2.333264000	-1.898133000
H	2.554125000	-3.615790000	0.221239000
H	3.082084000	-4.623928000	4.366945000
H	3.507321000	-5.096647000	1.963940000
H	4.346013000	2.115376000	-0.696827000
H	4.450658000	-1.435574000	-3.128137000
H	5.379186000	0.793517000	-2.536370000
H	-0.261259000	1.094110000	-2.494183000
H	-0.526010000	-3.720642000	0.812744000
H	-0.733667000	1.178183000	3.239119000
H	-1.242862000	-2.437797000	-3.927174000
H	-1.695679000	2.664756000	5.019073000
H	-2.029766000	-5.071592000	-0.616504000
H	-2.380727000	-1.200622000	0.529862000
H	-2.395062000	-4.434488000	-2.993637000
H	-2.499642000	2.333264000	-1.898133000
H	-2.554125000	3.615790000	0.221239000
H	-3.082084000	4.623928000	4.366945000
H	-3.507321000	5.096647000	1.963940000
H	-4.346013000	-2.115376000	-0.696827000
H	-4.450658000	1.435574000	-3.128137000
H	-5.379186000	-0.793517000	-2.536370000
P	0.870816000	-1.181154000	0.381364000
P	-0.870816000	1.181154000	0.381364000

B3LYP, NEUTRAL

C	0.000000000	0.000000000	1.036234000
C	0.176328000	2.421370000	-1.641053000
C	0.194759000	-2.166618000	-4.004923000
C	0.498902000	2.749261000	-2.948916000
C	0.852917000	-1.516273000	-1.367615000
C	0.873081000	-2.577445000	2.631418000
C	1.003496000	-3.732333000	3.391631000
C	1.206513000	-1.253185000	-3.742987000
C	1.277121000	-2.562755000	1.296900000
C	1.529710000	-4.887178000	2.823479000
C	1.535727000	-0.929669000	-2.432319000
C	1.801469000	-3.725642000	0.731574000
C	1.924384000	-4.882629000	1.490822000
C	2.823453000	-0.310220000	0.376685000
C	3.001191000	0.992158000	0.833843000
C	3.933452000	-1.041399000	-0.050048000
C	4.268602000	1.562171000	0.859504000

C	5.198860000	-0.472442000	-0.027037000
C	5.368395000	0.832008000	0.427716000
C	-0.176328000	-2.421370000	-1.641053000
C	-0.194759000	2.166618000	-4.004923000
C	-0.498902000	-2.749261000	-2.948916000
C	-0.852917000	1.516273000	-1.367615000
C	-0.873081000	2.577445000	2.631418000
C	-1.003496000	3.732333000	3.391631000
C	-1.206513000	1.253185000	-3.742987000
C	-1.277121000	2.562755000	1.296900000
C	-1.529710000	4.887178000	2.823479000
C	-1.535727000	0.929669000	-2.432319000
C	-1.801469000	3.725642000	0.731574000
C	-1.924384000	4.882629000	1.490822000
C	-2.823453000	0.310220000	0.376685000
C	-3.001191000	-0.992158000	0.833843000
C	-3.933452000	1.041399000	-0.050048000
C	-4.268602000	-1.562171000	0.859504000
C	-5.198860000	0.472442000	-0.027037000
C	-5.368395000	-0.832008000	0.427716000
H	0.058811000	2.419328000	-5.026110000
H	0.443668000	-1.677488000	3.051897000
H	0.688348000	-3.732732000	4.427162000
H	0.727693000	2.869485000	-0.824730000
H	1.292876000	3.458031000	-3.145487000
H	1.625198000	-5.789016000	3.414154000
H	1.737497000	-0.781755000	-4.559174000
H	2.094790000	-3.740965000	-0.309353000
H	2.132613000	1.540502000	1.175975000
H	2.320234000	-0.211194000	-2.242707000
H	2.323366000	-5.781812000	1.039534000
H	3.814907000	-2.056255000	-0.404032000
H	4.396683000	2.574830000	1.219713000
H	6.053771000	-1.046087000	-0.360925000
H	6.356327000	1.273507000	0.448594000
H	-0.058811000	-2.419328000	-5.026110000
H	-0.443668000	1.677488000	3.051897000
H	-0.688348000	3.732732000	4.427162000
H	-0.727693000	-2.869485000	-0.824730000
H	-1.292876000	-3.458031000	-3.145487000
H	-1.625198000	5.789016000	3.414154000
H	-1.737497000	0.781755000	-4.559174000
H	-2.094790000	3.740965000	-0.309353000
H	-2.132613000	-1.540502000	1.175975000
H	-2.320234000	0.211194000	-2.242707000
H	-2.323366000	5.781812000	1.039534000
H	-3.814907000	2.056255000	-0.404032000
H	-4.396683000	-2.574830000	1.219713000
H	-6.053771000	1.046087000	-0.360925000
H	-6.356327000	-1.273507000	0.448594000
P	1.112210000	-0.988774000	0.370956000

P	-1.112210000	0.988774000	0.370956000

OLYP, GROUND-STATE CATION

C	0.000000000	0.000000000	0.921239000
C	0.291718000	-1.735391000	-2.214382000
C	0.303798000	-1.836158000	-3.600684000
C	0.456867000	-4.029090000	2.784392000
C	0.551231000	-2.757062000	2.225058000
C	0.895184000	-5.145405000	2.067660000
C	1.082570000	-2.603640000	0.935262000
C	1.364651000	-1.126182000	-1.545822000
C	1.382837000	-1.330035000	-4.329763000
C	1.428982000	-4.990339000	0.786544000
C	1.519915000	-3.722232000	0.213468000
C	2.441431000	-0.611569000	-2.278332000
C	2.447378000	-0.718037000	-3.669157000
C	2.829057000	-0.235419000	0.802421000
C	2.887388000	1.100361000	1.218841000
C	3.985200000	-1.026592000	0.791086000
C	4.106818000	1.643716000	1.620781000
C	5.199995000	-0.474772000	1.193910000
C	5.261180000	0.858014000	1.608962000
C	-0.291718000	1.735391000	-2.214382000
C	-0.303798000	1.836158000	-3.600684000
C	-0.456867000	4.029090000	2.784392000
C	-0.551231000	2.757062000	2.225058000
C	-0.895184000	5.145405000	2.067660000
C	-1.082570000	2.603640000	0.935262000
C	-1.364651000	1.126182000	-1.545822000
C	-1.382837000	1.330035000	-4.329763000
C	-1.428982000	4.990339000	0.786544000
C	-1.519915000	3.722232000	0.213468000
C	-2.441431000	0.611569000	-2.278332000
C	-2.447378000	0.718037000	-3.669157000
C	-2.829057000	0.235419000	0.802421000
C	-2.887388000	-1.100361000	1.218841000
C	-3.985200000	1.026592000	0.791086000
C	-4.106818000	-1.643716000	1.620781000
C	-5.199995000	0.474772000	1.193910000
C	-5.261180000	-0.858014000	1.608962000
H	0.042392000	-4.149220000	3.782209000
H	0.215436000	-1.882314000	2.772712000
H	0.530006000	2.305565000	-4.115322000
H	0.549412000	2.127018000	-1.649623000
H	0.821045000	-6.136816000	2.507672000
H	1.389446000	-1.410183000	-5.413677000
H	1.770395000	-5.858505000	0.228613000
H	1.922027000	-3.604593000	-0.787353000
H	1.982891000	1.700722000	1.246698000
H	3.270303000	-0.134353000	-1.766807000
H	3.287043000	-0.322821000	-4.235187000

H	3.935070000	-2.065783000	0.479737000
H	4.154528000	2.678912000	1.949227000
H	6.097504000	-1.087805000	1.191623000
H	6.209338000	1.282385000	1.929028000
H	-0.042392000	4.149220000	3.782209000
H	-0.215436000	1.882314000	2.772712000
H	-0.530006000	-2.305565000	-4.115322000
H	-0.549412000	-2.127018000	-1.649623000
H	-0.821045000	6.136816000	2.507672000
H	-1.389446000	1.410183000	-5.413677000
H	-1.770395000	5.858505000	0.228613000
H	-1.922027000	3.604593000	-0.787353000
H	-1.982891000	-1.700722000	1.246698000
H	-3.270303000	0.134353000	-1.766807000
H	-3.287043000	0.322821000	-4.235187000
H	-3.935070000	2.065783000	0.479737000
H	-4.154528000	-2.678912000	1.949227000
H	-6.097504000	1.087805000	1.191623000
H	-6.209338000	-1.282385000	1.929028000
P	1.254487000	-0.933859000	0.251844000
P	-1.254487000	0.933859000	0.251844000

B3LYP, GROUND-STATE CATION

C	0.000000000	0.000000000	1.067447000
C	0.129802000	-2.165883000	-4.016168000
C	0.239726000	2.409850000	-1.652035000
C	0.577045000	2.727894000	-2.958364000
C	0.813878000	-1.526731000	-1.393645000
C	0.833448000	-2.559342000	2.630103000
C	0.943607000	-3.715192000	3.390702000
C	1.168257000	-1.279164000	-3.764352000
C	1.198565000	-2.577750000	1.282101000
C	1.419917000	-4.887329000	2.815896000
C	1.510465000	-0.955625000	-2.458407000
C	1.674630000	-3.758308000	0.705388000
C	1.785075000	-4.907895000	1.474245000
C	2.759726000	-0.247309000	0.384053000
C	2.884495000	1.064690000	0.839673000
C	3.892829000	-0.955197000	-0.022589000
C	4.134427000	1.668314000	0.875480000
C	5.138172000	-0.346796000	0.016957000
C	5.259461000	0.965098000	0.463685000
C	-0.129802000	2.165883000	-4.016168000
C	-0.239726000	-2.409850000	-1.652035000
C	-0.577045000	-2.727894000	-2.958364000
C	-0.813878000	1.526731000	-1.393645000
C	-0.833448000	2.559342000	2.630103000
C	-0.943607000	3.715192000	3.390702000
C	-1.168257000	1.279164000	-3.764352000
C	-1.198565000	2.577750000	1.282101000
C	-1.419917000	4.887329000	2.815896000

C	-1.510465000	0.955625000	-2.458407000
C	-1.674630000	3.758308000	0.705388000
C	-1.785075000	4.907895000	1.474245000
C	-2.759726000	0.247309000	0.384053000
C	-2.884495000	-1.064690000	0.839673000
C	-3.892829000	0.955197000	-0.022589000
C	-4.134427000	-1.668314000	0.875480000
C	-5.138172000	0.346796000	0.016957000
C	-5.259461000	-0.965098000	0.463685000
H	0.129709000	2.420176000	-5.034924000
H	0.463865000	-1.644236000	3.070450000
H	0.658023000	-3.699271000	4.433674000
H	0.789428000	2.857936000	-0.835123000
H	1.386346000	3.419062000	-3.150817000
H	1.505142000	-5.786049000	3.411734000
H	1.714880000	-0.835327000	-4.584949000
H	1.946638000	-3.788526000	-0.340541000
H	2.013490000	1.605871000	1.183340000
H	2.152866000	-5.820231000	1.024970000
H	2.315422000	-0.259163000	-2.274123000
H	3.808086000	-1.977152000	-0.366736000
H	4.229408000	2.684822000	1.232415000
H	6.014683000	-0.897062000	-0.296542000
H	6.232824000	1.435457000	0.496498000
H	-0.129709000	-2.420176000	-5.034924000
H	-0.463865000	1.644236000	3.070450000
H	-0.658023000	3.699271000	4.433674000
H	-0.789428000	-2.857936000	-0.835123000
H	-1.386346000	-3.419062000	-3.150817000
H	-1.505142000	5.786049000	3.411734000
H	-1.714880000	0.835327000	-4.584949000
H	-1.946638000	3.788526000	-0.340541000
H	-2.013490000	-1.605871000	1.183340000
H	-2.152866000	5.820231000	1.024970000
H	-2.315422000	0.259163000	-2.274123000
H	-3.808086000	1.977152000	-0.366736000
H	-4.229408000	-2.684822000	1.232415000
H	-6.014683000	0.897062000	-0.296542000
H	-6.232824000	-1.435457000	0.496498000
P	1.134249000	-1.042631000	0.326486000
P	-1.134249000	1.042631000	0.326486000

17. $Si(PPh_3)_2$, C_2

OLYP, NEUTRAL

C	0.149825000	2.229510000	-0.519084000
C	0.297825000	1.834151000	-1.854034000
C	0.966718000	3.257449000	-0.022693000
C	1.232498000	2.459713000	-2.679146000
C	1.769479000	-2.669809000	1.575822000
C	1.886144000	3.892062000	-0.851442000

C	2.025724000	3.492904000	-2.184503000
C	2.029314000	-3.903703000	0.963572000
C	2.226365000	-2.444506000	2.880479000
C	2.314566000	-0.725667000	-0.425565000
C	2.716577000	-4.901578000	1.652953000
C	2.824393000	-1.482161000	-1.489662000
C	2.914944000	-3.443908000	3.567300000
C	2.915938000	0.491168000	-0.097087000
C	3.161442000	-4.675433000	2.956893000
C	3.910508000	-1.011683000	-2.226118000
C	3.999609000	0.962262000	-0.836341000
C	4.498979000	0.214000000	-1.902126000
C	-0.149825000	-2.229510000	-0.519084000
C	-0.297825000	-1.834151000	-1.854034000
C	-0.966718000	-3.257449000	-0.022693000
C	-1.232498000	-2.459713000	-2.679146000
C	-1.769479000	2.669809000	1.575822000
C	-1.886144000	-3.892062000	-0.851442000
C	-2.025724000	-3.492904000	-2.184503000
C	-2.029314000	3.903703000	0.963572000
C	-2.226365000	2.444506000	2.880479000
C	-2.314566000	0.725667000	-0.425565000
C	-2.716577000	4.901578000	1.652953000
C	-2.824393000	1.482161000	-1.489662000
C	-2.914944000	3.443908000	3.567300000
C	-2.915938000	-0.491168000	-0.097087000
C	-3.161442000	4.675433000	2.956893000
C	-3.910508000	1.011683000	-2.226118000
C	-3.999609000	-0.962262000	-0.836341000
C	-4.498979000	-0.214000000	-1.902126000
H	0.327734000	-1.045680000	-2.252868000
H	0.875947000	3.554182000	1.018145000
H	1.342062000	2.133403000	-3.710993000
H	1.681191000	-4.090645000	-0.046233000
H	2.045447000	-1.484599000	3.357046000
H	2.370878000	-2.432617000	-1.748365000
H	2.502624000	4.696225000	-0.455487000
H	2.507255000	1.066674000	0.728935000
H	2.754763000	3.979283000	-2.828093000
H	2.908523000	-5.856178000	1.167442000
H	3.261548000	-3.257284000	4.581250000
H	3.701955000	-5.452920000	3.492125000
H	4.298494000	-1.601990000	-3.053862000
H	4.442342000	1.923026000	-0.588361000
H	5.341654000	0.583096000	-2.482855000
H	-0.327734000	1.045680000	-2.252868000
H	-0.875947000	-3.554182000	1.018145000
H	-1.342062000	-2.133403000	-3.710993000
H	-1.681191000	4.090645000	-0.046233000
H	-2.045447000	1.484599000	3.357046000
H	-2.370878000	2.432617000	-1.748365000

H	-2.502624000	-4.696225000	-0.455487000
H	-2.507255000	-1.066674000	0.728935000
H	-2.754763000	-3.979283000	-2.828093000
H	-2.908523000	5.856178000	1.167442000
H	-3.261548000	3.257284000	4.581250000
H	-3.701955000	5.452920000	3.492125000
H	-4.298494000	1.601990000	-3.053862000
H	-4.442342000	-1.923026000	-0.588361000
H	-5.341654000	-0.583096000	-2.482855000
P	0.935213000	-1.323696000	0.650812000
P	-0.935213000	1.323696000	0.650812000
Si	0.000000000	0.000000000	2.208799000

B3LYP, NEUTRAL

C	0.135928815	2.245207000	-0.549143895
C	0.292574746	1.843400609	-1.875922533
C	0.957634817	3.262874571	-0.054922865
C	1.240410911	2.448966015	-2.692165499
C	1.799024573	-2.665511299	1.575376648
C	1.889103610	3.878631763	-0.875121463
C	2.037281786	3.470950224	-2.197817863
C	2.040756007	-3.925345467	1.023673949
C	2.288993413	-2.381740100	2.851180057
C	2.356074936	-0.757944263	-0.454734637
C	2.743132165	-4.885143801	1.739895217
C	2.879212040	-1.527277283	-1.497555203
C	2.962562546	0.452005940	-0.133818273
C	2.990979271	-3.343518091	3.565434966
C	3.218940717	-4.598187104	3.013474764
C	3.979592502	-1.079534091	-2.214357430
C	4.063294626	0.900161243	-0.852762592
C	4.573315034	0.137694983	-1.894188926
C	-0.135928815	-2.245207000	-0.549143895
C	-0.292574746	-1.843400609	-1.875922533
C	-0.957634817	-3.262874571	-0.054922865
C	-1.240410911	-2.448966015	-2.692165499
C	-1.799024573	2.665511299	1.575376648
C	-1.889103610	-3.878631763	-0.875121463
C	-2.037281786	-3.470950224	-2.197817863
C	-2.040756007	3.925345467	1.023673949
C	-2.288993413	2.381740100	2.851180057
C	-2.356074936	0.757944263	-0.454734637
C	-2.743132165	4.885143801	1.739895217
C	-2.879212040	1.527277283	-1.497555203
C	-2.962562546	-0.452005940	-0.133818273
C	-2.990979271	3.343518091	3.565434966
C	-3.218940717	4.598187104	3.013474764
C	-3.979592502	1.079534091	-2.214357430
C	-4.063294626	-0.900161243	-0.852762592
C	-4.573315034	-0.137694983	-1.894188926
H	0.331003379	-1.059916320	-2.279560034

H	0.868769102	3.567003357	0.979388216
H	1.354738568	2.117383968	-3.715800392
H	1.675638939	-4.166340827	0.035627585
H	2.125143149	-1.404644312	3.286310974
H	2.429921312	-2.476263268	-1.754547899
H	2.508005981	4.673339808	-0.479417062
H	2.560537970	1.039600403	0.680839957
H	2.773610592	3.943618491	-2.833886401
H	2.919428234	-5.858034107	1.299910708
H	3.360775134	-3.109200499	4.554933499
H	3.767129014	-5.346581149	3.570419510
H	4.377096541	-1.682244976	-3.020696596
H	4.515210532	1.850078413	-0.601001510
H	5.430377703	0.486504910	-2.455449783
H	-0.331003379	1.059916320	-2.279560034
H	-0.868769102	-3.567003357	0.979388216
H	-1.354738568	-2.117383968	-3.715800392
H	-1.675638939	4.166340827	0.035627585
H	-2.125143149	1.404644312	3.286310974
H	-2.429921312	2.476263268	-1.754547899
H	-2.508005981	-4.673339808	-0.479417062
H	-2.560537970	-1.039600403	0.680839957
H	-2.773610592	-3.943618491	-2.833886401
H	-2.919428234	5.858034107	1.299910708
H	-3.360775134	3.109200499	4.554933499
H	-3.767129014	5.346581149	3.570419510
H	-4.377096541	1.682244976	-3.020696596
H	-4.515210532	-1.850078413	-0.601001510
H	-5.430377703	-0.486504910	-2.455449783
P	0.961354842	-1.341930981	0.616315904
P	-0.961354842	1.341930981	0.616315904
Si	0.000000000	0.000000000	2.164693001

OLYP, GROUND-STATE CATION

C	0.152407000	2.259737000	-0.552082000
C	0.307426000	1.855589000	-1.884117000
C	0.971180000	3.276824000	-0.035757000
C	1.257447000	2.473728000	-2.694511000
C	1.787375000	-2.730993000	1.553435000
C	1.913826000	3.893696000	-0.853383000
C	1.987848000	-4.007979000	1.008577000
C	2.059033000	3.494132000	-2.183572000
C	2.275824000	-2.438304000	2.834554000
C	2.355541000	-0.747574000	-0.458650000
C	2.657566000	-4.981494000	1.746033000
C	2.865652000	-1.483163000	-1.539079000
C	2.945049000	-3.417871000	3.567710000
C	2.956582000	0.460941000	-0.090797000
C	3.136033000	-4.689590000	3.025924000
C	3.955936000	-0.994979000	-2.254989000
C	4.050985000	0.939734000	-0.807089000

C	4.549975000	0.216211000	-1.889764000
C	-0.152407000	-2.259737000	-0.552082000
C	-0.307426000	-1.855589000	-1.884117000
C	-0.971180000	-3.276824000	-0.035757000
C	-1.257447000	-2.473728000	-2.694511000
C	-1.787375000	2.730993000	1.553435000
C	-1.913826000	-3.893696000	-0.853383000
C	-1.987848000	4.007979000	1.008577000
C	-2.059033000	-3.494132000	-2.183572000
C	-2.275824000	2.438304000	2.834554000
C	-2.355541000	0.747574000	-0.458650000
C	-2.657566000	4.981494000	1.746033000
C	-2.865652000	1.483163000	-1.539079000
C	-2.945049000	3.417871000	3.567710000
C	-2.956582000	-0.460941000	-0.090797000
C	-3.136033000	4.689590000	3.025924000
C	-3.955936000	0.994979000	-2.254989000
C	-4.050985000	-0.939734000	-0.807089000
C	-4.549975000	-0.216211000	-1.889764000
H	0.324308000	-1.075201000	-2.289482000
H	0.858069000	3.593711000	0.996404000
H	1.369101000	2.158212000	-3.728698000
H	1.615728000	-4.243806000	0.017512000
H	2.140645000	-1.450110000	3.265722000
H	2.414260000	-2.428976000	-1.818896000
H	2.535211000	4.689717000	-0.450724000
H	2.562443000	1.032131000	0.743073000
H	2.796228000	3.976899000	-2.819765000
H	2.808001000	-5.969927000	1.318922000
H	3.317127000	-3.184682000	4.562119000
H	3.658395000	-5.452033000	3.598366000
H	4.347229000	-1.564567000	-3.094276000
H	4.506497000	1.883875000	-0.523419000
H	5.402791000	0.592451000	-2.449238000
H	-0.324308000	1.075201000	-2.289482000
H	-0.858069000	-3.593711000	0.996404000
H	-1.369101000	-2.158212000	-3.728698000
H	-1.615728000	4.243806000	0.017512000
H	-2.140645000	1.450110000	3.265722000
H	-2.414260000	2.428976000	-1.818896000
H	-2.535211000	-4.689717000	-0.450724000
H	-2.562443000	-1.032131000	0.743073000
H	-2.796228000	-3.976899000	-2.819765000
H	-2.808001000	5.969927000	1.318922000
H	-3.317127000	3.184682000	4.562119000
H	-3.658395000	5.452033000	3.598366000
H	-4.347229000	1.564567000	-3.094276000
H	-4.506497000	-1.883875000	-0.523419000
H	-5.402791000	-0.592451000	-2.449238000
P	1.008234000	-1.418139000	0.559363000
P	-1.008234000	1.418139000	0.559363000

Si	0.000000000	0.000000000	2.125654000

B3LYP, GROUND-STATE CATION

C	0.510413000	-1.600277000	-3.474657000
C	0.560635000	-1.622846000	-1.066597000
C	0.657035000	2.310732000	-1.060021000
C	0.698127000	2.282069000	-3.463652000
C	1.137191000	-1.261063000	-2.282577000
C	1.284709000	2.632967000	-2.251958000
C	1.600696000	-4.010451000	0.721092000
C	1.704810000	-2.774992000	1.361137000
C	1.960318000	-5.171786000	1.393775000
C	2.181930000	-2.716065000	2.674988000
C	2.424271000	-5.109438000	2.701045000
C	2.537072000	-3.879533000	3.340066000
C	2.943927000	-0.461493000	0.206602000
C	3.258894000	0.814527000	0.667242000
C	3.911847000	-1.208485000	-0.473196000
C	4.518706000	1.351073000	0.431623000
C	5.164459000	-0.666533000	-0.713598000
C	5.468557000	0.615222000	-0.263388000
C	-0.510413000	1.600277000	-3.474657000
C	-0.560635000	1.622846000	-1.066597000
C	-0.657035000	-2.310732000	-1.060021000
C	-0.698127000	-2.282069000	-3.463652000
C	-1.137191000	1.261063000	-2.282577000
C	-1.284709000	-2.632967000	-2.251958000
C	-1.600696000	4.010451000	0.721092000
C	-1.704810000	2.774992000	1.361137000
C	-1.960318000	5.171786000	1.393775000
C	-2.181930000	2.716065000	2.674988000
C	-2.424271000	5.109438000	2.701045000
C	-2.537072000	3.879533000	3.340066000
C	-2.943927000	0.461493000	0.206602000
C	-3.258894000	-0.814527000	0.667242000
C	-3.911847000	1.208485000	-0.473196000
C	-4.518706000	-1.351073000	0.431623000
C	-5.164459000	0.666533000	-0.713598000
C	-5.468557000	-0.615222000	-0.263388000
H	0.966214000	-1.317598000	-4.413604000
H	1.108817000	2.606792000	-0.124084000
H	1.183655000	2.540904000	-4.394837000
H	1.245623000	-4.075124000	-0.296861000
H	1.879032000	-6.126156000	0.891451000
H	2.063297000	-0.707328000	-2.308119000
H	2.226903000	3.163432000	-2.235094000
H	2.288304000	-1.764875000	3.181169000
H	2.530687000	1.383477000	1.229009000
H	2.702432000	-6.016267000	3.220663000
H	2.903768000	-3.825444000	4.355973000
H	3.688986000	-2.212544000	-0.808550000

H	4.758902000	2.339309000	0.799556000
H	5.908029000	-1.247015000	-1.242525000
H	6.449878000	1.032562000	-0.444663000
H	-0.966214000	1.317598000	-4.413604000
H	-1.108817000	-2.606792000	-0.124084000
H	-1.183655000	-2.540904000	-4.394837000
H	-1.245623000	4.075124000	-0.296861000
H	-1.879032000	6.126156000	0.891451000
H	-2.063297000	0.707328000	-2.308119000
H	-2.226903000	-3.163432000	-2.235094000
H	-2.288304000	1.764875000	3.181169000
H	-2.530687000	-1.383477000	1.229009000
H	-2.702432000	6.016267000	3.220663000
H	-2.903768000	3.825444000	4.355973000
H	-3.688986000	2.212544000	-0.808550000
H	-4.758902000	-2.339309000	0.799556000
H	-5.908029000	1.247015000	-1.242525000
H	-6.449878000	-1.032562000	-0.444663000
P	1.312495000	-1.200630000	0.532630000
P	-1.312495000	1.200630000	0.532630000
Si	0.000000000	0.000000000	2.104433000

18. $Ge(PPh_3)_2$, C_2

OLYP, NEUTRAL

C	0.108930000	2.291157000	-0.542217000
C	0.265821000	1.896004000	-1.877000000
C	0.949715000	3.291079000	-0.027199000
C	1.233621000	2.496430000	-2.682944000
C	1.823684000	-2.686162000	1.584325000
C	1.896873000	3.904391000	-0.839410000
C	1.953763000	-4.000713000	1.114410000
C	2.046522000	3.505703000	-2.172140000
C	2.355388000	-0.792284000	-0.492520000
C	2.413167000	-2.339964000	2.810157000
C	2.630831000	-4.956624000	1.871647000
C	2.901553000	-1.591580000	-1.505836000
C	2.908209000	0.461678000	-0.225765000
C	3.088798000	-3.297896000	3.563975000
C	3.196571000	-4.611149000	3.100412000
C	3.981462000	-1.126794000	-2.254541000
C	3.986049000	0.926404000	-0.977569000
C	4.523955000	0.135212000	-1.992502000
C	-0.108930000	-2.291157000	-0.542217000
C	-0.265821000	-1.896004000	-1.877000000
C	-0.949715000	-3.291079000	-0.027199000
C	-1.233621000	-2.496430000	-2.682944000
C	-1.823684000	2.686162000	1.584325000
C	-1.896873000	-3.904391000	-0.839410000
C	-1.953763000	4.000713000	1.114410000
C	-2.046522000	-3.505703000	-2.172140000

C	-2.355388000	0.792284000	-0.492520000
C	-2.413167000	2.339964000	2.810157000
C	-2.630831000	4.956624000	1.871647000
C	-2.901553000	1.591580000	-1.505836000
C	-2.908209000	-0.461678000	-0.225765000
C	-3.088798000	3.297896000	3.563975000
C	-3.196571000	4.611149000	3.100412000
C	-3.981462000	1.126794000	-2.254541000
C	-3.986049000	-0.926404000	-0.977569000
C	-4.523955000	-0.135212000	-1.992502000
Ge	0.000000000	0.000000000	2.208988000
H	0.369755000	-1.119215000	-2.285490000
H	0.859965000	3.576284000	1.016871000
H	1.354469000	2.167780000	-3.712693000
H	1.515685000	-4.281551000	0.163093000
H	2.344280000	-1.317406000	3.171871000
H	2.472919000	1.065195000	0.566011000
H	2.479042000	-2.569937000	-1.711749000
H	2.531229000	4.687264000	-0.429177000
H	2.717985000	-5.974709000	1.497914000
H	2.801861000	3.971157000	-2.800517000
H	3.533370000	-3.015690000	4.515874000
H	3.724847000	-5.357776000	3.689038000
H	4.394223000	1.913314000	-0.778643000
H	4.403237000	-1.749818000	-3.040560000
H	5.364800000	0.497655000	-2.580001000
H	-0.369755000	1.119215000	-2.285490000
H	-0.859965000	-3.576284000	1.016871000
H	-1.354469000	-2.167780000	-3.712693000
H	-1.515685000	4.281551000	0.163093000
H	-2.344280000	1.317406000	3.171871000
H	-2.472919000	-1.065195000	0.566011000
H	-2.479042000	2.569937000	-1.711749000
H	-2.531229000	-4.687264000	-0.429177000
H	-2.717985000	5.974709000	1.497914000
H	-2.801861000	-3.971157000	-2.800517000
H	-3.533370000	3.015690000	4.515874000
H	-3.724847000	5.357776000	3.689038000
H	-4.394223000	-1.913314000	-0.778643000
H	-4.403237000	1.749818000	-3.040560000
H	-5.364800000	-0.497655000	-2.580001000
P	0.993005000	-1.383017000	0.600638000
P	-0.993005000	1.383017000	0.600638000

B3LYP, NEUTRAL

C	0.104441000	2.252813000	-0.547560000
C	0.244407000	1.843165000	-1.873726000
C	0.949609000	3.256232000	-0.062521000
C	1.199882000	2.426309000	-2.697936000
C	1.833060000	-2.722751000	1.566859000
C	1.886852000	3.850962000	-0.891081000

C	2.019680000	3.434100000	-2.212987000
C	2.040844000	-3.989839000	1.017961000
C	2.347751000	-2.443510000	2.834732000
C	2.393501000	-0.780837000	-0.439324000
C	2.732718000	-4.960858000	1.729681000
C	2.934977000	-1.548307000	-1.474470000
C	2.973752000	0.443753000	-0.125746000
C	3.039116000	-3.415956000	3.543966000
C	3.232298000	-4.678140000	2.994952000
C	4.029133000	-1.084752000	-2.190670000
C	4.068940000	0.907047000	-0.844040000
C	4.597404000	0.146527000	-1.877462000
C	-0.104441000	-2.252813000	-0.547560000
C	-0.244407000	-1.843165000	-1.873726000
C	-0.949609000	-3.256232000	-0.062521000
C	-1.199882000	-2.426309000	-2.697936000
C	-1.833060000	2.722751000	1.566859000
C	-1.886852000	-3.850962000	-0.891081000
C	-2.019680000	-3.434100000	-2.212987000
C	-2.040844000	3.989839000	1.017961000
C	-2.347751000	2.443510000	2.834732000
C	-2.393501000	0.780837000	-0.439324000
C	-2.732718000	4.960858000	1.729681000
C	-2.934977000	1.548307000	-1.474470000
C	-2.973752000	-0.443753000	-0.125746000
C	-3.039116000	3.415956000	3.543966000
C	-3.232298000	4.678140000	2.994952000
C	-4.029133000	1.084752000	-2.190670000
C	-4.068940000	-0.907047000	-0.844040000
C	-4.597404000	-0.146527000	-1.877462000
Ge	0.000000000	0.000000000	2.278590000
H	0.395682000	-1.069150000	-2.270069000
H	0.874944000	3.563668000	0.972054000
H	1.301789000	2.087855000	-3.720678000
H	1.656560000	-4.227330000	0.036289000
H	2.215813000	-1.459602000	3.265897000
H	2.503669000	-2.507439000	-1.725634000
H	2.524477000	4.634325000	-0.502232000
H	2.556499000	1.028625000	0.683952000
H	2.762462000	3.888334000	-2.855061000
H	2.882897000	-5.939359000	1.292151000
H	3.428365000	-3.184700000	4.526742000
H	3.772676000	-5.435213000	3.547919000
H	4.441850000	-1.685766000	-2.990761000
H	4.501781000	1.867403000	-0.598232000
H	5.450305000	0.507275000	-2.437690000
H	-0.395682000	1.069150000	-2.270069000
H	-0.874944000	-3.563668000	0.972054000
H	-1.301789000	-2.087855000	-3.720678000
H	-1.656560000	4.227330000	0.036289000
H	-2.215813000	1.459602000	3.265897000

H	-2.503669000	2.507439000	-1.725634000
H	-2.524477000	-4.634325000	-0.502232000
H	-2.556499000	-1.028625000	0.683952000
H	-2.762462000	-3.888334000	-2.855061000
H	-2.882897000	5.939359000	1.292151000
H	-3.428365000	3.184700000	4.526742000
H	-3.772676000	5.435213000	3.547919000
H	-4.441850000	1.685766000	-2.990761000
H	-4.501781000	-1.867403000	-0.598232000
H	-5.450305000	-0.507275000	-2.437690000
P	1.005293000	-1.381486000	0.627687000
P	-1.005293000	1.381486000	0.627687000

OLYP, GROUND-STATE CATION

C	0.136863000	2.331503000	-0.529783000
C	0.316278000	1.940722000	-1.863288000
C	0.964879000	3.324282000	0.019099000
C	1.295965000	2.552286000	-2.642904000
C	1.850773000	-2.775331000	1.551546000
C	1.937025000	3.934727000	-0.768082000
C	1.968911000	-4.092076000	1.083519000
C	2.103965000	3.551257000	-2.100558000
C	2.366578000	-0.813650000	-0.509859000
C	2.428086000	-2.423251000	2.781385000
C	2.639230000	-5.045430000	1.847862000
C	2.881818000	-1.567266000	-1.575565000
C	2.933554000	0.424818000	-0.190599000
C	3.099312000	-3.381394000	3.540020000
C	3.202005000	-4.694259000	3.077169000
C	3.945293000	-1.068741000	-2.323913000
C	4.004553000	0.912330000	-0.936299000
C	4.509245000	0.169853000	-2.003477000
C	-0.136863000	-2.331503000	-0.529783000
C	-0.316278000	-1.940722000	-1.863288000
C	-0.964879000	-3.324282000	0.019099000
C	-1.295965000	-2.552286000	-2.642904000
C	-1.850773000	2.775331000	1.551546000
C	-1.937025000	-3.934727000	-0.768082000
C	-1.968911000	4.092076000	1.083519000
C	-2.103965000	-3.551257000	-2.100558000
C	-2.366578000	0.813650000	-0.509859000
C	-2.428086000	2.423251000	2.781385000
C	-2.639230000	5.045430000	1.847862000
C	-2.881818000	1.567266000	-1.575565000
C	-2.933554000	-0.424818000	-0.190599000
C	-3.099312000	3.381394000	3.540020000
C	-3.202005000	4.694259000	3.077169000
C	-3.945293000	1.068741000	-2.323913000
C	-4.004553000	-0.912330000	-0.936299000
C	-4.509245000	-0.169853000	-2.003477000
Ge	0.000000000	0.000000000	2.180236000

H	0.313565000	-1.169789000	-2.290979000
H	0.838206000	3.625828000	1.054306000
H	1.428437000	2.246203000	-3.677418000
H	1.530466000	-4.375207000	0.132787000
H	2.364355000	-1.403248000	3.151032000
H	2.453694000	-2.534505000	-1.817903000
H	2.531190000	1.011139000	0.628569000
H	2.565861000	4.711096000	-0.339263000
H	2.721917000	-6.065782000	1.481576000
H	2.865745000	4.027239000	-2.712541000
H	3.539100000	-3.101029000	4.493848000
H	3.721134000	-5.441957000	3.671636000
H	4.341798000	-1.652065000	-3.151272000
H	4.435584000	1.877481000	-0.687993000
H	5.344031000	0.551967000	-2.585887000
H	-0.313565000	1.169789000	-2.290979000
H	-0.838206000	-3.625828000	1.054306000
H	-1.428437000	-2.246203000	-3.677418000
H	-1.530466000	4.375207000	0.132787000
H	-2.364355000	1.403248000	3.151032000
H	-2.453694000	2.534505000	-1.817903000
H	-2.531190000	-1.011139000	0.628569000
H	-2.565861000	-4.711096000	-0.339263000
H	-2.721917000	6.065782000	1.481576000
H	-2.865745000	-4.027239000	-2.712541000
H	-3.539100000	3.101029000	4.493848000
H	-3.721134000	5.441957000	3.671636000
H	-4.341798000	1.652065000	-3.151272000
H	-4.435584000	-1.877481000	-0.687993000
H	-5.344031000	-0.551967000	-2.585887000
P	1.050355000	-1.484387000	0.545376000
P	-1.050355000	1.484387000	0.545376000

B3LYP, GROUND-STATE CATION

C	0.469956000	-1.619449000	-3.439262000
C	0.570953000	-1.642835000	-1.032074000
C	0.657168000	2.311486000	-1.000736000
C	0.745815000	2.287333000	-3.403213000
C	1.125883000	-1.288677000	-2.260541000
C	1.312243000	2.628739000	-2.179551000
C	1.604806000	-4.057875000	0.736295000
C	1.767236000	-2.821742000	1.361393000
C	1.963170000	-5.224002000	1.401596000
C	2.296273000	-2.767689000	2.655452000
C	2.484701000	-5.165787000	2.687061000
C	2.652879000	-3.934867000	3.312636000
C	2.987509000	-0.500420000	0.193514000
C	3.305309000	0.777395000	0.647408000
C	3.943947000	-1.240324000	-0.509865000
C	4.555377000	1.322988000	0.382026000
C	5.186914000	-0.689716000	-0.779408000

C	5.492692000	0.594160000	-0.337092000
C	-0.469956000	1.619449000	-3.439262000
C	-0.570953000	1.642835000	-1.032074000
C	-0.657168000	-2.311486000	-1.000736000
C	-0.745815000	-2.287333000	-3.403213000
C	-1.125883000	1.288677000	-2.260541000
C	-1.312243000	-2.628739000	-2.179551000
C	-1.604806000	4.057875000	0.736295000
C	-1.767236000	2.821742000	1.361393000
C	-1.963170000	5.224002000	1.401596000
C	-2.296273000	2.767689000	2.655452000
C	-2.484701000	5.165787000	2.687061000
C	-2.652879000	3.934867000	3.312636000
C	-2.987509000	0.500420000	0.193514000
C	-3.305309000	-0.777395000	0.647408000
C	-3.943947000	1.240324000	-0.509865000
C	-4.555377000	-1.322988000	0.382026000
C	-5.186914000	0.689716000	-0.779408000
C	-5.492692000	-0.594160000	-0.337092000
Ge	0.000000000	0.000000000	2.221486000
H	0.909880000	-1.343028000	-4.387601000
H	1.095347000	2.596420000	-0.054659000
H	1.206699000	-4.119066000	-0.266047000
H	1.253291000	2.541052000	-4.324120000
H	1.836949000	-6.178938000	0.909488000
H	2.059109000	-0.748400000	-2.306212000
H	2.261577000	3.145284000	-2.143167000
H	2.447468000	-1.815532000	3.149174000
H	2.584723000	1.342510000	1.222593000
H	2.762518000	-6.076127000	3.200665000
H	3.063345000	-3.883997000	4.311848000
H	3.718995000	-2.245163000	-0.841686000
H	4.797162000	2.313266000	0.743478000
H	5.921349000	-1.264379000	-1.327210000
H	6.466004000	1.018422000	-0.543462000
H	-0.909880000	1.343028000	-4.387601000
H	-1.095347000	-2.596420000	-0.054659000
H	-1.206699000	4.119066000	-0.266047000
H	-1.253291000	-2.541052000	-4.324120000
H	-1.836949000	6.178938000	0.909488000
H	-2.059109000	0.748400000	-2.306212000
H	-2.261577000	-3.145284000	-2.143167000
H	-2.447468000	1.815532000	3.149174000
H	-2.584723000	-1.342510000	1.222593000
H	-2.762518000	6.076127000	3.200665000
H	-3.063345000	3.883997000	4.311848000
H	-3.718995000	2.245163000	-0.841686000
H	-4.797162000	-2.313266000	0.743478000
H	-5.921349000	1.264379000	-1.327210000
H	-6.466004000	-1.018422000	-0.543462000
P	1.363619000	-1.239588000	0.552793000

P	-1.363619000	1.239588000	0.552793000

19.Sn(PPh$_3$)$_2$, C_2

OLYP, NEUTRAL

C	0.029191000	2.317850000	-0.509510000
C	0.164856000	1.901295000	-1.840473000
C	0.900329000	3.301933000	-0.013508000
C	1.142199000	2.466642000	-2.661238000
C	1.853912000	3.882773000	-0.842086000
C	1.955630000	-2.809186000	1.555188000
C	1.982712000	3.463124000	-2.170719000
C	1.992754000	-4.131885000	1.090880000
C	2.434788000	-0.817885000	-0.441413000
C	2.665618000	-2.477465000	2.721355000
C	2.697839000	-5.106776000	1.796526000
C	2.924842000	0.466326000	-0.196548000
C	3.028425000	-1.613903000	-1.430871000
C	3.366972000	-3.455040000	3.424151000
C	3.383762000	-4.774888000	2.966787000
C	3.993334000	0.960971000	-0.943571000
C	4.100690000	-1.120373000	-2.170944000
C	4.585184000	0.169101000	-1.927600000
C	-0.029191000	-2.317850000	-0.509510000
C	-0.164856000	-1.901295000	-1.840473000
C	-0.900329000	-3.301933000	-0.013508000
C	-1.142199000	-2.466642000	-2.661238000
C	-1.853912000	-3.882773000	-0.842086000
C	-1.955630000	2.809186000	1.555188000
C	-1.982712000	-3.463124000	-2.170719000
C	-1.992754000	4.131885000	1.090880000
C	-2.434788000	0.817885000	-0.441413000
C	-2.665618000	2.477465000	2.721355000
C	-2.697839000	5.106776000	1.796526000
C	-2.924842000	-0.466326000	-0.196548000
C	-3.028425000	1.613903000	-1.430871000
C	-3.366972000	3.455040000	3.424151000
C	-3.383762000	4.774888000	2.966787000
C	-3.993334000	-0.960971000	-0.943571000
C	-4.100690000	1.120373000	-2.170944000
C	-4.585184000	-0.169101000	-1.927600000
H	0.491239000	-1.133207000	-2.233043000
H	0.829834000	3.598382000	1.028980000
H	1.248068000	2.120687000	-3.686985000
H	1.464031000	-4.403116000	0.184101000
H	2.457487000	1.068641000	0.578270000
H	2.511339000	4.654105000	-0.446753000
H	2.650333000	-2.613921000	-1.620364000
H	2.675306000	-1.448640000	3.072907000
H	2.713549000	-6.130209000	1.427433000
H	2.744624000	3.900955000	-2.810867000

H	3.905170000	-3.183276000	4.329568000
H	3.933853000	-5.537132000	3.513881000
H	4.353956000	1.969417000	-0.761523000
H	4.562970000	-1.742471000	-2.934656000
H	5.422579000	0.552691000	-2.506617000
H	-0.491239000	1.133207000	-2.233043000
H	-0.829834000	-3.598382000	1.028980000
H	-1.248068000	-2.120687000	-3.686985000
H	-1.464031000	4.403116000	0.184101000
H	-2.457487000	-1.068641000	0.578270000
H	-2.511339000	-4.654105000	-0.446753000
H	-2.650333000	2.613921000	-1.620364000
H	-2.675306000	1.448640000	3.072907000
H	-2.713549000	6.130209000	1.427433000
H	-2.744624000	-3.900955000	-2.810867000
H	-3.905170000	3.183276000	4.329568000
H	-3.933853000	5.537132000	3.513881000
H	-4.353956000	-1.969417000	-0.761523000
H	-4.562970000	1.742471000	-2.934656000
H	-5.422579000	-0.552691000	-2.506617000
P	1.099975000	-1.467533000	0.649804000
P	-1.099975000	1.467533000	0.649804000
Sn	0.000000000	0.000000000	2.503424000

B3LYP, NEUTRAL

C	0.043632000	2.244187000	-0.531887000
C	0.129583000	1.797913000	-1.850860000
C	0.953879000	3.209431000	-0.088382000
C	1.101108000	2.305020000	-2.707023000
C	1.901338000	-2.879381000	1.542460000
C	1.901739000	3.734171000	-0.951163000
C	1.966010000	-4.164004000	0.999615000
C	1.984740000	3.277394000	-2.264062000
C	2.490490000	-0.850366000	-0.366143000
C	2.542345000	-2.627205000	2.758519000
C	2.641361000	-5.176989000	1.668514000
C	3.011669000	0.410403000	-0.098318000
C	3.086237000	-1.644446000	-1.349940000
C	3.215129000	-3.641334000	3.424951000
C	3.265949000	-4.920502000	2.882930000
C	4.107126000	0.881972000	-0.812352000
C	4.180885000	-1.173957000	-2.059384000
C	4.693398000	0.092843000	-1.791273000
C	-0.043632000	-2.244187000	-0.531887000
C	-0.129583000	-1.797913000	-1.850860000
C	-0.953879000	-3.209431000	-0.088382000
C	-1.101108000	-2.305020000	-2.707023000
C	-1.901338000	2.879381000	1.542460000
C	-1.901739000	-3.734171000	-0.951163000
C	-1.966010000	4.164004000	0.999615000
C	-1.984740000	-3.277394000	-2.264062000

C	-2.490490000	0.850366000	-0.366143000
C	-2.542345000	2.627205000	2.758519000
C	-2.641361000	5.176989000	1.668514000
C	-3.011669000	-0.410403000	-0.098318000
C	-3.086237000	1.644446000	-1.349940000
C	-3.215129000	3.641334000	3.424951000
C	-3.265949000	4.920502000	2.882930000
C	-4.107126000	-0.881972000	-0.812352000
C	-4.180885000	1.173957000	-2.059384000
C	-4.693398000	-0.092843000	-1.791273000
H	0.562124000	-1.053032000	-2.214770000
H	0.920999000	3.541484000	0.940809000
H	1.162347000	1.937802000	-3.723083000
H	1.485513000	-4.381908000	0.056688000
H	2.525718000	-1.630403000	3.181473000
H	2.555541000	1.015946000	0.674551000
H	2.589538000	4.489780000	-0.594499000
H	2.681269000	-6.168324000	1.236137000
H	2.695910000	-2.630574000	-1.562180000
H	2.739032000	3.672473000	-2.931238000
H	3.702399000	-3.429639000	4.367641000
H	3.792382000	-5.710943000	3.401467000
H	4.495622000	1.869496000	-0.603917000
H	4.638724000	-1.796074000	-2.817638000
H	5.548600000	0.458745000	-2.344511000
H	-0.562124000	1.053032000	-2.214770000
H	-0.920999000	-3.541484000	0.940809000
H	-1.162347000	-1.937802000	-3.723083000
H	-1.485513000	4.381908000	0.056688000
H	-2.525718000	1.630403000	3.181473000
H	-2.555541000	-1.015946000	0.674551000
H	-2.589538000	-4.489780000	-0.594499000
H	-2.681269000	6.168324000	1.236137000
H	-2.695910000	2.630574000	-1.562180000
H	-2.739032000	-3.672473000	-2.931238000
H	-3.702399000	3.429639000	4.367641000
H	-3.792382000	5.710943000	3.401467000
H	-4.495622000	-1.869496000	-0.603917000
H	-4.638724000	1.796074000	-2.817638000
H	-5.548600000	-0.458745000	-2.344511000
P	1.098407000	-1.472633000	0.679586000
P	-1.098407000	1.472633000	0.679586000
Sn	0.000000000	0.000000000	2.579077000

OLYP, GROUND-STATE CATION

C	0.069199000	2.374932000	-0.472042000
C	0.254557000	1.968040000	-1.800001000
C	0.911793000	3.357242000	0.074170000
C	1.249921000	2.557473000	-2.577846000
C	1.899137000	3.945917000	-0.710949000
C	1.985915000	-2.899021000	1.529458000

C	2.030130000	-4.220189000	1.061593000
C	2.068428000	3.549707000	-2.039545000
C	2.438392000	-0.871261000	-0.480420000
C	2.657988000	-2.564965000	2.716001000
C	2.720599000	-5.193346000	1.783274000
C	2.961995000	0.394794000	-0.197541000
C	2.962343000	-1.625786000	-1.541866000
C	3.350690000	-3.541064000	3.430539000
C	3.378571000	-4.858025000	2.968564000
C	3.993743000	-1.104011000	-2.318048000
C	4.002894000	0.905681000	-0.970753000
C	4.517505000	0.160539000	-2.030979000
C	-0.069199000	-2.374932000	-0.472042000
C	-0.254557000	-1.968040000	-1.800001000
C	-0.911793000	-3.357242000	0.074170000
C	-1.249921000	-2.557473000	-2.577846000
C	-1.899137000	-3.945917000	-0.710949000
C	-1.985915000	2.899021000	1.529458000
C	-2.030130000	4.220189000	1.061593000
C	-2.068428000	-3.549707000	-2.039545000
C	-2.438392000	0.871261000	-0.480420000
C	-2.657988000	2.564965000	2.716001000
C	-2.720599000	5.193346000	1.783274000
C	-2.961995000	-0.394794000	-0.197541000
C	-2.962343000	1.625786000	-1.541866000
C	-3.350690000	3.541064000	3.430539000
C	-3.378571000	4.858025000	2.968564000
C	-3.993743000	1.104011000	-2.318048000
C	-4.002894000	-0.905681000	-0.970753000
C	-4.517505000	-0.160539000	-2.030979000
H	0.383842000	-1.203002000	-2.226266000
H	0.782619000	3.671098000	1.105532000
H	1.385152000	2.239130000	-3.608202000
H	1.520794000	-4.491453000	0.143557000
H	2.536671000	4.716197000	-0.284231000
H	2.551186000	0.985796000	0.614587000
H	2.564514000	-2.612130000	-1.758058000
H	2.657850000	-1.540844000	3.081226000
H	2.745648000	-6.216582000	1.416618000
H	2.840567000	4.009681000	-2.650546000
H	3.866504000	-3.272086000	4.348910000
H	3.913976000	-5.620200000	3.528971000
H	4.397500000	-1.688359000	-3.141083000
H	4.399941000	1.891705000	-0.750343000
H	5.327701000	0.561031000	-2.635319000
H	-0.383842000	1.203002000	-2.226266000
H	-0.782619000	-3.671098000	1.105532000
H	-1.385152000	-2.239130000	-3.608202000
H	-1.520794000	4.491453000	0.143557000
H	-2.536671000	-4.716197000	-0.284231000
H	-2.551186000	-0.985796000	0.614587000

H	-2.564514000	2.612130000	-1.758058000
H	-2.657850000	1.540844000	3.081226000
H	-2.745648000	6.216582000	1.416618000
H	-2.840567000	-4.009681000	-2.650546000
H	-3.866504000	3.272086000	4.348910000
H	-3.913976000	5.620200000	3.528971000
H	-4.397500000	1.688359000	-3.141083000
H	-4.399941000	-1.891705000	-0.750343000
H	-5.327701000	-0.561031000	-2.635319000
P	1.153797000	-1.570057000	0.599457000
P	-1.153797000	1.570057000	0.599457000
Sn	0.000000000	0.000000000	2.467682000

B3LYP, GROUND-STATE CATION

C	0.323324000	-1.651507000	-3.325055000
C	0.546489000	-1.678573000	-0.925776000
C	0.712429000	2.280315000	-0.830688000
C	0.924544000	2.250058000	-3.224994000
C	1.055804000	-1.356093000	-2.182334000
C	1.445021000	2.559516000	-1.972852000
C	1.654580000	-4.155391000	0.692137000
C	1.870717000	-2.943589000	1.347077000
C	2.047027000	-5.347160000	1.290186000
C	2.482505000	-2.940001000	2.605066000
C	2.654337000	-5.338204000	2.538629000
C	2.872709000	-4.131760000	3.195679000
C	3.049389000	-0.604103000	0.185739000
C	3.399393000	0.668758000	0.630049000
C	3.964670000	-1.345833000	-0.568830000
C	4.639434000	1.206520000	0.308586000
C	5.196937000	-0.802580000	-0.896399000
C	5.534838000	0.475376000	-0.459988000
C	-0.323324000	1.651507000	-3.325055000
C	-0.546489000	1.678573000	-0.925776000
C	-0.712429000	-2.280315000	-0.830688000
C	-0.924544000	-2.250058000	-3.224994000
C	-1.055804000	1.356093000	-2.182334000
C	-1.445021000	-2.559516000	-1.972852000
C	-1.654580000	4.155391000	0.692137000
C	-1.870717000	2.943589000	1.347077000
C	-2.047027000	5.347160000	1.290186000
C	-2.482505000	2.940001000	2.605066000
C	-2.654337000	5.338204000	2.538629000
C	-2.872709000	4.131760000	3.195679000
C	-3.049389000	0.604103000	0.185739000
C	-3.399393000	-0.668758000	0.630049000
C	-3.964670000	1.345833000	-0.568830000
C	-4.639434000	-1.206520000	0.308586000
C	-5.196937000	0.802580000	-0.896399000
C	-5.534838000	-0.475376000	-0.459988000
H	0.728856000	-1.400640000	-4.295617000

H	1.115720000	2.541507000	0.138052000
H	1.188646000	-4.177201000	-0.282426000
H	1.492503000	2.474304000	-4.117749000
H	1.880646000	-6.283177000	0.774228000
H	2.013093000	-0.866835000	-2.277740000
H	2.419812000	3.019436000	-1.886019000
H	2.676476000	-2.006237000	3.119318000
H	2.711533000	1.238201000	1.239883000
H	2.958886000	-6.267956000	2.999551000
H	3.349014000	-4.119459000	4.166504000
H	3.716352000	-2.347227000	-0.894200000
H	4.906054000	2.192796000	0.663623000
H	5.898918000	-1.378942000	-1.483553000
H	6.500227000	0.893766000	-0.711047000
H	-0.728856000	1.400640000	-4.295617000
H	-1.115720000	-2.541507000	0.138052000
H	-1.188646000	4.177201000	-0.282426000
H	-1.492503000	-2.474304000	-4.117749000
H	-1.880646000	6.283177000	0.774228000
H	-2.013093000	0.866835000	-2.277740000
H	-2.419812000	-3.019436000	-1.886019000
H	-2.676476000	2.006237000	3.119318000
H	-2.711533000	-1.238201000	1.239883000
H	-2.958886000	6.267956000	2.999551000
H	-3.349014000	4.119459000	4.166504000
H	-3.716352000	2.347227000	-0.894200000
H	-4.906054000	-2.192796000	0.663623000
H	-5.898918000	1.378942000	-1.483553000
H	-6.500227000	-0.893766000	-0.711047000
P	1.436194000	-1.327697000	0.621795000
P	-1.436194000	1.327697000	0.621795000
Sn	0.000000000	0.000000000	2.522845000

20. $Pb(PPh_3)_2$, C_2

OLYP, NEUTRAL

C	0.000466000	-2.382785000	-0.479344000
C	0.138874000	1.974819000	-1.812815000
C	0.884190000	3.345806000	0.033255000
C	1.131987000	2.531503000	-2.620613000
C	1.856258000	3.915923000	-0.781599000
C	1.986345000	3.507759000	-2.113835000
C	2.010540000	-2.884927000	1.559059000
C	2.036313000	-4.212279000	1.106427000
C	2.469547000	-0.902184000	-0.456488000
C	2.742605000	-2.545757000	2.710198000
C	2.756157000	-5.181476000	1.804638000
C	2.915364000	0.409517000	-0.282619000
C	3.087988000	-1.728250000	-1.406171000
C	3.457286000	-3.517739000	3.406761000
C	3.466344000	-4.841051000	2.957998000

C	3.967285000	0.898837000	-1.056966000
C	4.142385000	-1.239356000	-2.174315000
C	4.585017000	0.076080000	-1.999056000
C	-0.000466000	2.382785000	-0.479344000
C	-0.138874000	-1.974819000	-1.812815000
C	-0.884190000	-3.345806000	0.033255000
C	-1.131987000	-2.531503000	-2.620613000
C	-1.856258000	-3.915923000	-0.781599000
C	-1.986345000	-3.507759000	-2.113835000
C	-2.010540000	2.884927000	1.559059000
C	-2.036313000	4.212279000	1.106427000
C	-2.469547000	0.902184000	-0.456488000
C	-2.742605000	2.545757000	2.710198000
C	-2.756157000	5.181476000	1.804638000
C	-2.915364000	-0.409517000	-0.282619000
C	-3.087988000	1.728250000	-1.406171000
C	-3.457286000	3.517739000	3.406761000
C	-3.466344000	4.841051000	2.957998000
C	-3.967285000	-0.898837000	-1.056966000
C	-4.142385000	1.239356000	-2.174315000
C	-4.585017000	-0.076080000	-1.999056000
H	0.525686000	-1.219440000	-2.217007000
H	0.810688000	3.634087000	1.078061000
H	1.240279000	2.193302000	-3.648712000
H	1.487291000	-4.490230000	0.213528000
H	2.432221000	1.038662000	0.460428000
H	2.525773000	4.670040000	-0.373250000
H	2.740665000	-2.747970000	-1.542245000
H	2.760488000	3.938119000	-2.744168000
H	2.761934000	-1.513348000	3.052232000
H	2.763313000	-6.207994000	1.443964000
H	4.012322000	-3.239391000	4.300026000
H	4.027390000	-5.599268000	3.499460000
H	4.293905000	1.926661000	-0.928428000
H	4.624363000	-1.884610000	-2.905943000
H	5.411164000	0.454991000	-2.596999000
H	-0.525686000	1.219440000	-2.217007000
H	-0.810688000	-3.634087000	1.078061000
H	-1.240279000	-2.193302000	-3.648712000
H	-1.487291000	4.490230000	0.213528000
H	-2.432221000	-1.038662000	0.460428000
H	-2.525773000	-4.670040000	-0.373250000
H	-2.740665000	2.747970000	-1.542245000
H	-2.760488000	-3.938119000	-2.744168000
H	-2.761934000	1.513348000	3.052232000
H	-2.763313000	6.207994000	1.443964000
H	-4.012322000	3.239391000	4.300026000
H	-4.027390000	5.599268000	3.499460000
H	-4.293905000	-1.926661000	-0.928428000
H	-4.624363000	1.884610000	-2.905943000
H	-5.411164000	-0.454991000	-2.596999000

P	1.153916000	-1.540993000	0.661355000
P	-1.153916000	1.540993000	0.661355000
Pb	0.000000000	0.000000000	2.590872000

B3LYP, NEUTRAL

C	0.011363000	2.262503000	-0.520529000
C	0.088464000	1.812224000	-1.838671000
C	0.938544000	3.213597000	-0.080142000
C	1.062304000	2.307636000	-2.699424000
C	1.888819000	3.726726000	-0.947559000
C	1.952514000	-2.931745000	1.531732000
C	1.953790000	-4.233770000	1.027929000
C	1.958132000	3.271233000	-2.261682000
C	2.526452000	-0.885238000	-0.358438000
C	2.631278000	-5.245610000	1.696085000
C	2.660802000	-2.663068000	2.707167000
C	3.013256000	0.395161000	-0.121732000
C	3.145552000	-1.688306000	-1.320734000
C	3.321724000	-4.972234000	2.870255000
C	3.336078000	-3.676065000	3.372692000
C	4.100423000	0.876093000	-0.842787000
C	4.232102000	-1.208921000	-2.036297000
C	4.711831000	0.076568000	-1.797666000
C	-0.011363000	-2.262503000	-0.520529000
C	-0.088464000	-1.812224000	-1.838671000
C	-0.938544000	-3.213597000	-0.080142000
C	-1.062304000	-2.307636000	-2.699424000
C	-1.888819000	-3.726726000	-0.947559000
C	-1.952514000	2.931745000	1.531732000
C	-1.953790000	4.233770000	1.027929000
C	-1.958132000	-3.271233000	-2.261682000
C	-2.526452000	0.885238000	-0.358438000
C	-2.631278000	5.245610000	1.696085000
C	-2.660802000	2.663068000	2.707167000
C	-3.013256000	-0.395161000	-0.121732000
C	-3.145552000	1.688306000	-1.320734000
C	-3.321724000	4.972234000	2.870255000
C	-3.336078000	3.676065000	3.372692000
C	-4.100423000	-0.876093000	-0.842787000
C	-4.232102000	1.208921000	-2.036297000
C	-4.711831000	-0.076568000	-1.797666000
H	0.610985000	-1.072144000	-2.198537000
H	0.918096000	3.542118000	0.950633000
H	1.115761000	1.937601000	-3.714912000
H	1.422911000	-4.465492000	0.115820000
H	2.539232000	1.008859000	0.634002000
H	2.588762000	4.472495000	-0.593678000
H	2.620681000	-6.250660000	1.294537000
H	2.695292000	-1.652621000	3.096844000
H	2.713245000	3.658254000	-2.932645000
H	2.780112000	-2.689081000	-1.508469000

H	3.849349000	-5.762516000	3.387730000
H	3.875813000	-3.451130000	4.283173000
H	4.463435000	1.877805000	-0.657445000
H	4.709906000	-1.838132000	-2.776154000
H	5.561662000	0.448533000	-2.355154000
H	-0.610985000	1.072144000	-2.198537000
H	-0.918096000	-3.542118000	0.950633000
H	-1.115761000	-1.937601000	-3.714912000
H	-1.422911000	4.465492000	0.115820000
H	-2.539232000	-1.008859000	0.634002000
H	-2.588762000	-4.472495000	-0.593678000
H	-2.620681000	6.250660000	1.294537000
H	-2.695292000	1.652621000	3.096844000
H	-2.713245000	-3.658254000	-2.932645000
H	-2.780112000	2.689081000	-1.508469000
H	-3.849349000	5.762516000	3.387730000
H	-3.875813000	3.451130000	4.283173000
H	-4.463435000	-1.877805000	-0.657445000
H	-4.709906000	1.838132000	-2.776154000
H	-5.561662000	-0.448533000	-2.355154000
P	1.144457000	-1.514763000	0.691782000
P	-1.144457000	1.514763000	0.691782000
Pb	0.000000000	0.000000000	2.689069000

OLYP, GROUND-STATE CATION

C	0.103853000	3.316065000	-1.513845000
C	0.104503000	2.986025000	-0.152954000
C	1.077296000	4.177216000	-2.021254000
C	1.086533000	3.526811000	0.691963000
C	1.848241000	0.021806000	-1.368064000
C	2.043937000	4.399977000	0.182931000
C	2.045222000	4.721753000	-1.176844000
C	2.118048000	-4.267083000	1.637625000
C	2.118581000	-1.239113000	-0.823145000
C	2.243606000	-2.874654000	1.557646000
C	2.573437000	0.477824000	-2.467102000
C	3.019684000	-5.002398000	2.408549000
C	3.132266000	-2.037145000	-1.374774000
C	3.277000000	-2.227093000	2.255274000
C	3.571496000	-0.323555000	-3.024783000
C	3.851714000	-1.578120000	-2.476104000
C	4.046129000	-4.357261000	3.098795000
C	4.175949000	-2.968455000	3.017834000
C	-0.103853000	-3.316065000	-1.513845000
C	-0.104503000	-2.986025000	-0.152954000
C	-1.077296000	-4.177216000	-2.021254000
C	-1.086533000	-3.526811000	0.691963000
C	-1.848241000	-0.021806000	-1.368064000
C	-2.043937000	-4.399977000	0.182931000
C	-2.045222000	-4.721753000	-1.176844000
C	-2.118048000	4.267083000	1.637625000

C	-2.118581000	1.239113000	-0.823145000
C	-2.243606000	2.874654000	1.557646000
C	-2.573437000	-0.477824000	-2.467102000
C	-3.019684000	5.002398000	2.408549000
C	-3.132266000	2.037145000	-1.374774000
C	-3.277000000	2.227093000	2.255274000
C	-3.571496000	0.323555000	-3.024783000
C	-3.851714000	1.578120000	-2.476104000
C	-4.046129000	4.357261000	3.098795000
C	-4.175949000	2.968455000	3.017834000
H	0.650140000	-2.900332000	-2.173372000
H	1.073021000	4.427552000	-3.079151000
H	1.086948000	0.655522000	-0.930799000
H	1.099122000	3.270384000	1.748430000
H	1.325433000	-4.775444000	1.099716000
H	2.364232000	1.461793000	-2.875997000
H	2.794379000	4.823580000	0.845753000
H	2.799679000	5.395599000	-1.574574000
H	2.919621000	-6.083441000	2.465791000
H	3.354748000	-3.007495000	-0.941755000
H	3.387585000	-1.147150000	2.188706000
H	4.140457000	0.032623000	-3.879921000
H	4.637286000	-2.197086000	-2.902320000
H	4.745896000	-4.934233000	3.698036000
H	4.976819000	-2.462749000	3.551510000
H	-0.650140000	2.900332000	-2.173372000
H	-1.073021000	-4.427552000	-3.079151000
H	-1.086948000	-0.655522000	-0.930799000
H	-1.099122000	-3.270384000	1.748430000
H	-1.325433000	4.775444000	1.099716000
H	-2.364232000	-1.461793000	-2.875997000
H	-2.794379000	-4.823580000	0.845753000
H	-2.799679000	-5.395599000	-1.574574000
H	-2.919621000	6.083441000	2.465791000
H	-3.354748000	3.007495000	-0.941755000
H	-3.387585000	1.147150000	2.188706000
H	-4.140457000	-0.032623000	-3.879921000
H	-4.637286000	2.197086000	-2.902320000
H	-4.745896000	4.934233000	3.698036000
H	-4.976819000	2.462749000	3.551510000
P	1.119386000	-1.846434000	0.563252000
P	-1.119386000	1.846434000	0.563252000
Pb	0.000000000	0.000000000	2.374415000

B3LYP, GROUND-STATE CATION

C	0.354559000	-1.640564000	-3.290087000
C	0.602321000	-1.667372000	-0.893204000
C	0.666484000	2.244970000	-0.784470000
C	0.902838000	2.216327000	-3.176145000
C	1.104893000	-1.357770000	-2.155566000
C	1.415805000	2.514080000	-1.918146000

C	1.735542000	-4.167774000	0.658913000
C	1.967790000	-2.967320000	1.329063000
C	2.138762000	-5.370188000	1.228336000
C	2.606710000	-2.986676000	2.573357000
C	2.772354000	-5.383575000	2.463526000
C	3.007600000	-4.188577000	3.135625000
C	3.116692000	-0.600401000	0.198445000
C	3.448343000	0.684474000	0.622062000
C	4.042495000	-1.338970000	-0.546340000
C	4.680221000	1.235816000	0.291792000
C	5.267085000	-0.783279000	-0.881889000
C	5.586463000	0.505957000	-0.465417000
C	-0.354559000	1.640564000	-3.290087000
C	-0.602321000	1.667372000	-0.893204000
C	-0.666484000	-2.244970000	-0.784470000
C	-0.902838000	-2.216327000	-3.176145000
C	-1.104893000	1.357770000	-2.155566000
C	-1.415805000	-2.514080000	-1.918146000
C	-1.735542000	4.167774000	0.658913000
C	-1.967790000	2.967320000	1.329063000
C	-2.138762000	5.370188000	1.228336000
C	-2.606710000	2.986676000	2.573357000
C	-2.772354000	5.383575000	2.463526000
C	-3.007600000	4.188577000	3.135625000
C	-3.116692000	0.600401000	0.198445000
C	-3.448343000	-0.684474000	0.622062000
C	-4.042495000	1.338970000	-0.546340000
C	-4.680221000	-1.235816000	0.291792000
C	-5.267085000	0.783279000	-0.881889000
C	-5.586463000	-0.505957000	-0.465417000
H	0.754148000	-1.398223000	-4.265326000
H	1.065116000	2.494357000	0.189621000
H	1.247902000	-4.171871000	-0.305122000
H	1.484707000	2.430315000	-4.062382000
H	1.959189000	-6.297512000	0.701107000
H	2.071246000	-0.888710000	-2.261534000
H	2.398654000	2.954245000	-1.820033000
H	2.752121000	1.253156000	1.223302000
H	2.813106000	-2.061155000	3.098114000
H	3.084530000	-6.321667000	2.901902000
H	3.504790000	-4.193569000	4.096036000
H	3.808696000	-2.348789000	-0.856035000
H	4.932816000	2.230827000	0.632452000
H	5.977494000	-1.358765000	-1.459666000
H	6.546273000	0.933710000	-0.721830000
H	-0.754148000	1.398223000	-4.265326000
H	-1.065116000	-2.494357000	0.189621000
H	-1.247902000	4.171871000	-0.305122000
H	-1.484707000	-2.430315000	-4.062382000
H	-1.959189000	6.297512000	0.701107000
H	-2.071246000	0.888710000	-2.261534000

H	-2.398654000	-2.954245000	-1.820033000
H	-2.752121000	-1.253156000	1.223302000
H	-2.813106000	2.061155000	3.098114000
H	-3.084530000	6.321667000	2.901902000
H	-3.504790000	4.193569000	4.096036000
H	-3.808696000	2.348789000	-0.856035000
H	-4.932816000	-2.230827000	0.632452000
H	-5.977494000	1.358765000	-1.459666000
H	-6.546273000	-0.933710000	-0.721830000
P	1.511942000	-1.336544000	0.648267000
P	-1.511942000	1.336544000	0.648267000
Pb	0.000000000	0.000000000	2.627059000

21. CIm_2, C_2

OLYP, NEUTRAL

C	0.000000000	0.000000000	1.121309000
C	0.486700000	1.077525000	0.444566000
C	0.791071000	2.815755000	-1.054430000
C	1.465844000	3.132208000	0.077831000
C	-0.486700000	-1.077525000	0.444566000
C	-0.791071000	-2.815755000	-1.054430000
C	-1.465844000	-3.132208000	0.077831000
H	0.503477000	-1.164102000	-1.413728000
H	0.684250000	3.365854000	-1.977078000
H	1.587679000	2.057573000	1.928828000
H	2.060924000	4.001402000	0.310990000
H	-0.503477000	1.164102000	-1.413728000
H	-0.684250000	-3.365854000	-1.977078000
H	-1.587679000	-2.057573000	1.928828000
H	-2.060924000	-4.001402000	0.310990000
N	0.256116000	1.541891000	-0.870403000
N	1.314653000	2.073874000	0.959780000
N	-0.256116000	-1.541891000	-0.870403000
N	-1.314653000	-2.073874000	0.959780000

B3LYP, NEUTRAL

C	0.000000000	0.000000000	1.064595000
C	0.473563000	1.091444000	0.417946000
C	0.754220000	2.870235000	-1.023040000
C	1.476246000	3.130602000	0.081452000
C	-0.473563000	-1.091444000	0.417946000
C	-0.754220000	-2.870235000	-1.023040000
C	-1.476246000	-3.130602000	0.081452000
H	0.572534000	-1.248275000	-1.404417000
H	0.618982000	3.455659000	-1.913822000
H	1.663581000	1.982411000	1.882157000
H	2.090598000	3.979434000	0.318649000
H	-0.572534000	1.248275000	-1.404417000
H	-0.618982000	-3.455659000	-1.913822000
H	-1.663581000	-1.982411000	1.882157000

H	-2.090598000	-3.979434000	0.318649000
N	0.191403000	1.605759000	-0.859841000
N	1.336361000	2.047894000	0.935628000
N	-0.191403000	-1.605759000	-0.859841000
N	-1.336361000	-2.047894000	0.935628000

OLYP, GROUND-STATE CATION

C	0.000000000	0.000000000	0.851066000
C	0.000000000	0.682413000	-1.215965000
C	0.000000000	2.487469000	0.600212000
C	0.000000000	-0.682413000	-1.215965000
C	0.000000000	-2.487469000	0.600212000
H	0.000000000	1.394425000	-2.027742000
H	0.000000000	3.147775000	-0.267342000
H	0.000000000	-1.394425000	-2.027742000
H	0.000000000	-3.147775000	-0.267342000
H	0.897392000	2.658527000	1.199888000
H	0.897392000	-2.658527000	1.199888000
H	-0.897392000	2.658527000	1.199888000
H	-0.897392000	-2.658527000	1.199888000
N	0.000000000	1.099562000	0.111808000
N	0.000000000	-1.099562000	0.111808000

B3LYP, GROUND-STATE CATION

C	0.000000000	0.000000000	0.961912000
C	0.000000000	0.713098000	-1.197750000
C	0.000000000	2.466944000	0.580029000
C	0.000000000	-0.713098000	-1.197750000
C	0.000000000	-2.466944000	0.580029000
H	0.000000000	1.386474000	-2.040925000
H	0.000000000	3.159107000	-0.256532000
H	0.000000000	-1.386474000	-2.040925000
H	0.000000000	-3.159107000	-0.256532000
H	0.886368000	2.611594000	1.193124000
H	0.886368000	-2.611594000	1.193124000
H	-0.886368000	2.611594000	1.193124000
H	-0.886368000	-2.611594000	1.193124000
N	0.000000000	1.088817000	0.074255000
N	0.000000000	-1.088817000	0.074255000

22. $SiIm_2$, C_2

OLYP, NEUTRAL

C	0.638790000	1.228770000	0.586114000
C	0.958474000	2.702089000	-1.160132000
C	1.589463000	3.217307000	-0.077462000
C	-0.638790000	-1.228770000	0.586114000
C	-0.958474000	-2.702089000	-1.160132000
C	-1.589463000	-3.217307000	-0.077462000
H	0.346858000	-1.035112000	-1.244953000
H	0.873260000	3.082612000	-2.166589000

H	1.729070000	2.447894000	1.906484000
H	2.160940000	4.126200000	0.034717000
H	-0.346858000	1.035112000	-1.244953000
H	-0.873260000	-3.082612000	-2.166589000
H	-1.729070000	-2.447894000	1.906484000
H	-2.160940000	-4.126200000	0.034717000
N	0.435549000	1.465934000	-0.775676000
N	1.421650000	2.314573000	0.955171000
N	-0.435549000	-1.465934000	-0.775676000
N	-1.421650000	-2.314573000	0.955171000
Si	0.000000000	0.000000000	1.818674000

B3LYP, NEUTRAL

C	0.609841000	1.236865000	0.615369000
C	0.911197000	2.668976000	-1.160434000
C	1.572265000	3.189740000	-0.110670000
C	-0.609841000	-1.236865000	0.615369000
C	-0.911197000	-2.668976000	-1.160434000
C	-1.572265000	-3.189740000	-0.110670000
H	0.409725000	-1.013791000	-1.194970000
H	0.810059000	3.029725000	-2.167733000
H	1.777710000	2.439866000	1.872005000
H	2.159048000	4.086732000	-0.035440000
H	-0.409725000	1.013791000	-1.194970000
H	-0.810059000	-3.029725000	-2.167733000
H	-1.777710000	-2.439866000	1.872005000
H	-2.159048000	-4.086732000	-0.035440000
N	0.367747000	1.456917000	-0.736169000
N	1.404856000	2.315564000	0.947144000
N	-0.367747000	-1.456917000	-0.736169000
N	-1.404856000	-2.315564000	0.947144000
Si	0.000000000	0.000000000	1.875815000

OLYP, GROUND-STATE CATION

C	0.612703000	1.268057000	0.583264000
C	0.786631000	2.782274000	-1.111796000
C	1.654328000	3.162127000	-0.133119000
C	-0.612703000	-1.268057000	0.583264000
C	-0.786631000	-2.782274000	-1.111796000
C	-1.654328000	-3.162127000	-0.133119000
H	0.558077000	3.231361000	-2.066061000
H	0.589274000	-1.161629000	-1.126394000
H	2.050579000	2.252559000	1.745765000
H	2.333613000	3.998879000	-0.076778000
H	-0.558077000	-3.231361000	-2.066061000
H	-0.589274000	1.161629000	-1.126394000
H	-2.050579000	-2.252559000	1.745765000
H	-2.333613000	-3.998879000	-0.076778000
N	0.175318000	1.627774000	-0.660870000
N	1.533316000	2.232357000	0.876054000
N	-0.175318000	-1.627774000	-0.660870000

N	-1.533316000	-2.232357000	0.876054000
Si	0.000000000	0.000000000	1.873891000

B3LYP, GROUND-STATE CATION

C	0.578351000	1.282252000	0.607863000
C	0.732573000	2.757455000	-1.111021000
C	1.621085000	3.140340000	-0.166453000
C	-0.578351000	-1.282252000	0.607863000
C	-0.732573000	-2.757455000	-1.111021000
C	-1.621085000	-3.140340000	-0.166453000
H	0.494194000	3.188347000	-2.066107000
H	0.658759000	-1.155514000	-1.068946000
H	2.053078000	2.255483000	1.713808000
H	2.310965000	3.964030000	-0.145509000
H	-0.494194000	-3.188347000	-2.066107000
H	-0.658759000	1.155514000	-1.068946000
H	-2.053078000	-2.255483000	1.713808000
H	-2.310965000	-3.964030000	-0.145509000
N	0.115526000	1.620833000	-0.625009000
N	1.509247000	2.232905000	0.864487000
N	-0.115526000	-1.620833000	-0.625009000
N	-1.509247000	-2.232905000	0.864487000
Si	0.000000000	0.000000000	1.927798000

23.GeIm$_2$, C_2

OLYP, NEUTRAL

C	0.617911000	1.274211000	0.654140000
C	0.844406000	2.667500000	-1.174182000
C	1.550722000	3.219958000	-0.157350000
C	-0.617911000	-1.274211000	0.654140000
C	-0.844406000	-2.667500000	-1.174182000
C	-1.550722000	-3.219958000	-0.157350000
Ge	0.000000000	0.000000000	2.029275000
H	0.460194000	-0.994382000	-1.118832000
H	0.701906000	3.001450000	-2.190766000
H	1.835657000	2.516887000	1.833695000
H	2.144531000	4.120625000	-0.126026000
H	-0.460194000	0.994382000	-1.118832000
H	-0.701906000	-3.001450000	-2.190766000
H	-1.835657000	-2.516887000	1.833695000
H	-2.144531000	-4.120625000	-0.126026000
N	0.321971000	1.468016000	-0.694514000
N	1.424113000	2.367284000	0.926207000
N	-0.321971000	-1.468016000	-0.694514000
N	-1.424113000	-2.367284000	0.926207000

B3LYP, NEUTRAL

C	0.619872000	1.288293000	0.642483000
C	0.857422000	2.688664000	-1.165382000
C	1.576076000	3.214333000	-0.155829000

C	-0.619872000	-1.288293000	0.642483000
C	-0.857422000	-2.688664000	-1.165382000
C	-1.576076000	-3.214333000	-0.155829000
Ge	0.000000000	0.000000000	2.014862000
H	0.467004000	-1.031135000	-1.121438000
H	0.712254000	3.035231000	-2.172063000
H	1.873868000	2.481431000	1.819810000
H	2.179653000	4.102420000	-0.120266000
H	-0.467004000	1.031135000	-1.121438000
H	-0.712254000	-3.035231000	-2.172063000
H	-1.873868000	-2.481431000	1.819810000
H	-2.179653000	-4.102420000	-0.120266000
N	0.310382000	1.499645000	-0.690011000
N	1.440083000	2.356685000	0.922275000
N	-0.310382000	-1.499645000	-0.690011000
N	-1.440083000	-2.356685000	0.922275000

OLYP, GROUND-STATE CATION

C	0.632484000	1.307779000	0.658613000
C	0.731155000	2.722964000	-1.125710000
C	1.673336000	3.126425000	-0.231757000
C	-0.632484000	-1.307779000	0.658613000
C	-0.731155000	-2.722964000	-1.125710000
C	-1.673336000	-3.126425000	-0.231757000
Ge	0.000000000	0.000000000	2.102809000
H	0.451736000	3.123681000	-2.088152000
H	0.678282000	-1.137212000	-0.957462000
H	2.181064000	2.299532000	1.657888000
H	2.379400000	3.941761000	-0.269191000
H	-0.451736000	-3.123681000	-2.088152000
H	-0.678282000	1.137212000	-0.957462000
H	-2.181064000	-2.299532000	1.657888000
H	-2.379400000	-3.941761000	-0.269191000
N	0.118379000	1.616807000	-0.564705000
N	1.593321000	2.253900000	0.836081000
N	-0.118379000	-1.616807000	-0.564705000
N	-1.593321000	-2.253900000	0.836081000

B3LYP, GROUND-STATE CATION

C	0.619019000	1.330170000	0.644191000
C	0.728372000	2.761038000	-1.113040000
C	1.683582000	3.129726000	-0.230765000
C	-0.619019000	-1.330170000	0.644191000
C	-0.728372000	-2.761038000	-1.113040000
C	-1.683582000	-3.129726000	-0.230765000
Ge	0.000000000	0.000000000	2.097225000
H	0.449620000	3.178323000	-2.063309000
H	0.708825000	-1.204618000	-0.954217000
H	2.195016000	2.262147000	1.638104000
H	2.403190000	3.926742000	-0.267298000
H	-0.449620000	-3.178323000	-2.063309000

H	-0.708825000	1.204618000	-0.954217000
H	-2.195016000	-2.262147000	1.638104000
H	-2.403190000	-3.926742000	-0.267298000
N	0.098389000	1.660360000	-0.561571000
N	1.597331000	2.245275000	0.826304000
N	-0.098389000	-1.660360000	-0.561571000
N	-1.597331000	-2.245275000	0.826304000

24. $SnIm_2$, C_2

OLYP, NEUTRAL

C	0.660310000	1.371773000	0.725390000
C	0.780822000	2.665360000	-1.183506000
C	1.612942000	3.222345000	-0.267744000
C	-0.660310000	-1.371773000	0.725390000
C	-0.780822000	-2.665360000	-1.183506000
C	-1.612942000	-3.222345000	-0.267744000
H	0.552904000	2.961113000	-2.196241000
H	0.556780000	-1.026918000	-0.940517000
H	2.066374000	2.580872000	1.709834000
H	2.250414000	4.090460000	-0.334180000
H	-0.552904000	-2.961113000	-2.196241000
H	-0.556780000	1.026918000	-0.940517000
H	-2.066374000	-2.580872000	1.709834000
H	-2.250414000	-4.090460000	-0.334180000
N	0.233837000	1.538598000	-0.583121000
N	1.537048000	2.429103000	0.865803000
N	-0.233837000	-1.538598000	-0.583121000
N	-1.537048000	-2.429103000	0.865803000
Sn	0.000000000	0.000000000	2.342586000

B3LYP, NEUTRAL

C	0.648489000	1.395606000	0.707423000
C	0.767019000	2.711585000	-1.169278000
C	1.626160000	3.226941000	-0.267353000
C	-0.648489000	-1.395606000	0.707423000
C	-0.767019000	-2.711585000	-1.169278000
C	-1.626160000	-3.226941000	-0.267353000
H	0.525932000	3.031663000	-2.166131000
H	0.597971000	-1.096370000	-0.933503000
H	2.110662000	2.523687000	1.680929000
H	2.279598000	4.077130000	-0.332124000
H	-0.525932000	-3.031663000	-2.166131000
H	-0.597971000	1.096370000	-0.933503000
H	-2.110662000	-2.523687000	1.680929000
H	-2.279598000	-4.077130000	-0.332124000
N	0.196881000	1.594836000	-0.574885000
N	1.549694000	2.417751000	0.854021000
N	-0.196881000	-1.594836000	-0.574885000
N	-1.549694000	-2.417751000	0.854021000
Sn	0.000000000	0.000000000	2.335820000

OLYP, GROUND-STATE CATION

C	0.683556000	1.389641000	0.737033000
C	0.699346000	2.689457000	-1.139680000
C	1.724517000	3.104991000	-0.348436000
C	-0.683556000	-1.389641000	0.737033000
C	-0.699346000	-2.689457000	-1.139680000
C	-1.724517000	-3.104991000	-0.348436000
H	0.361279000	3.039687000	-2.103118000
H	0.741695000	-1.168275000	-0.769735000
H	2.345888000	2.369842000	1.543248000
H	2.457419000	3.884475000	-0.490080000
H	-0.361279000	-3.039687000	-2.103118000
H	-0.741695000	1.168275000	-0.769735000
H	-2.345888000	-2.369842000	1.543248000
H	-2.457419000	-3.884475000	-0.490080000
N	0.089462000	1.649909000	-0.459088000
N	1.691287000	2.300266000	0.776053000
N	-0.089462000	-1.649909000	-0.459088000
N	-1.691287000	-2.300266000	0.776053000
Sn	0.000000000	0.000000000	2.441628000

B3LYP, GROUND-STATE CATION

C	0.659242000	1.427166000	0.721540000
C	0.687159000	2.746069000	-1.126406000
C	1.724920000	3.121280000	-0.347338000
C	-0.659242000	-1.427166000	0.721540000
C	-0.687159000	-2.746069000	-1.126406000
C	-1.724920000	-3.121280000	-0.347338000
H	0.348919000	3.118762000	-2.075884000
H	0.787291000	-1.260837000	-0.761882000
H	2.349220000	2.341342000	1.523989000
H	2.470868000	3.881728000	-0.488218000
H	-0.348919000	-3.118762000	-2.075884000
H	-0.787291000	1.260837000	-0.761882000
H	-2.349220000	-2.341342000	1.523989000
H	-2.470868000	-3.881728000	-0.488218000
N	0.058733000	1.712543000	-0.455501000
N	1.686213000	2.303135000	0.765966000
N	-0.058733000	-1.712543000	-0.455501000
N	-1.686213000	-2.303135000	0.765966000
Sn	0.000000000	0.000000000	2.421491000

25. $PbIm_2$, C_2

OLYP, NEUTRAL

C	0.662303000	1.437647000	0.754825000
C	0.711215000	2.687127000	-1.185407000
C	1.584583000	3.261148000	-0.318500000
C	-0.662303000	-1.437647000	0.754825000
C	-0.711215000	-2.687127000	-1.185407000

C	-1.584583000	-3.261148000	-0.318500000
H	0.441082000	2.961563000	-2.193751000
H	0.609053000	-1.047755000	-0.854679000
H	2.124048000	2.652016000	1.647378000
H	2.223023000	4.123650000	-0.431635000
H	-0.441082000	-2.961563000	-2.193751000
H	-0.609053000	1.047755000	-0.854679000
H	-2.124048000	-2.652016000	1.647378000
H	-2.223023000	-4.123650000	-0.431635000
N	0.180495000	1.583477000	-0.532040000
N	1.549037000	2.492146000	0.835512000
N	-0.180495000	-1.583477000	-0.532040000
N	-1.549037000	-2.492146000	0.835512000
Pb	0.000000000	0.000000000	2.490616000

B3LYP, NEUTRAL

C	0.643217000	2.654645000	-1.157864000
C	0.659133000	1.448111000	0.790112000
C	1.658281000	3.129139000	-0.405302000
C	-0.643217000	-2.654645000	-1.157864000
C	-0.659133000	-1.448111000	0.790112000
C	-1.658281000	-3.129139000	-0.405302000
H	0.296642000	2.952028000	-2.130407000
H	0.788878000	-1.151874000	-0.665664000
H	2.332409000	2.463767000	1.500574000
H	2.364875000	3.914588000	-0.599917000
H	-0.296642000	-2.952028000	-2.130407000
H	-0.788878000	1.151874000	-0.665664000
H	-2.332409000	-2.463767000	1.500574000
H	-2.364875000	-3.914588000	-0.599917000
N	0.053622000	1.641874000	-0.419939000
N	1.651830000	2.386913000	0.765590000
N	-0.053622000	-1.641874000	-0.419939000
N	-1.651830000	-2.386913000	0.765590000
Pb	0.000000000	0.000000000	2.579653000

OLYP, GROUND-STATE CATION

C	0.668042000	2.704038000	-1.146774000
C	0.699918000	1.464112000	0.771965000
C	1.715381000	3.140700000	-0.398105000
C	-0.668042000	-2.704038000	-1.146774000
C	-0.699918000	-1.464112000	0.771965000
C	-1.715381000	-3.140700000	-0.398105000
H	0.304405000	3.024612000	-2.111022000
H	0.761702000	-1.195614000	-0.689619000
H	2.386863000	2.460978000	1.497477000
H	2.446435000	3.912328000	-0.584289000
H	-0.304405000	-3.024612000	-2.111022000
H	-0.761702000	1.195614000	-0.689619000
H	-2.386863000	-2.460978000	1.497477000
H	-2.446435000	-3.912328000	-0.584289000

N	0.074609000	1.689629000	-0.414150000
N	1.709522000	2.371227000	0.752898000
N	-0.074609000	-1.689629000	-0.414150000
N	-1.709522000	-2.371227000	0.752898000
Pb	0.000000000	0.000000000	2.577259000

B3LYP, GROUND-STATE CATION

C	0.640584000	2.797038000	-1.120817000
C	0.664684000	1.511020000	0.751926000
C	1.731196000	3.148357000	-0.405914000
C	-0.640584000	-2.797038000	-1.120817000
C	-0.664684000	-1.511020000	0.751926000
C	-1.731196000	-3.148357000	-0.405914000
H	0.265031000	3.164536000	-2.058102000
H	0.856450000	-1.363441000	-0.653655000
H	2.425217000	2.377270000	1.444220000
H	2.494292000	3.879312000	-0.600037000
H	-0.265031000	-3.164536000	-2.058102000
H	-0.856450000	1.363441000	-0.653655000
H	-2.425217000	-2.377270000	1.444220000
H	-2.494292000	-3.879312000	-0.600037000
N	0.013121000	1.799509000	-0.394982000
N	1.721362000	2.350481000	0.724148000
N	-0.013121000	-1.799509000	-0.394982000
N	-1.721362000	-2.350481000	0.724148000
Pb	0.000000000	0.000000000	2.560449000

26.PAr, C_{2v}

OLYP, NEUTRAL

P	0.000000000	0.000000000	1.104835000
C	0.000000000	0.000000000	-0.637933000
C	0.000000000	0.000000000	-3.448111000
C	1.218640000	0.000000000	-2.755604000
C	1.229806000	0.000000000	-1.356648000
C	2.351346000	0.000000000	-0.473204000
C	2.999913000	0.000000000	1.852640000
C	-1.218640000	0.000000000	-2.755604000
C	-1.229806000	0.000000000	-1.356648000
C	-2.351346000	0.000000000	-0.473204000
C	-2.999913000	0.000000000	1.852640000
H	0.000000000	0.000000000	-4.535351000
H	2.156736000	0.000000000	-3.308658000
H	2.481253000	0.000000000	2.816520000
H	3.391034000	0.000000000	-0.808212000
H	3.639492000	0.891480000	1.796054000
H	3.639492000	-0.891480000	1.796054000
H	-2.156736000	0.000000000	-3.308658000
H	-2.481253000	0.000000000	2.816520000
H	-3.391034000	0.000000000	-0.808212000
H	-3.639492000	0.891480000	1.796054000

H	-3.639492000	-0.891480000	1.796054000
N	2.013562000	0.000000000	0.791216000
N	-2.013562000	0.000000000	0.791216000

B3LYP, NEUTRAL

P	0.000000000	0.000000000	1.113854000
C	0.000000000	0.000000000	-0.626396000
C	0.000000000	0.000000000	-3.428674000
C	1.211275000	0.000000000	-2.737152000
C	1.222860000	0.000000000	-1.344807000
C	2.350426000	0.000000000	-0.473781000
C	3.035566000	0.000000000	1.836861000
C	-1.211275000	0.000000000	-2.737152000
C	-1.222860000	0.000000000	-1.344807000
C	-2.350426000	0.000000000	-0.473781000
C	-3.035566000	0.000000000	1.836861000
H	0.000000000	0.000000000	-4.509703000
H	2.145330000	0.000000000	-3.285706000
H	2.530335000	0.000000000	2.801248000
H	3.380133000	0.000000000	-0.824572000
H	3.670495000	0.886964000	1.779349000
H	3.670495000	-0.886964000	1.779349000
H	-2.145330000	0.000000000	-3.285706000
H	-2.530335000	0.000000000	2.801248000
H	-3.380133000	0.000000000	-0.824572000
H	-3.670495000	0.886964000	1.779349000
H	-3.670495000	-0.886964000	1.779349000
N	2.036205000	0.000000000	0.786552000
N	-2.036205000	0.000000000	0.786552000

OLYP, GROUND-STATE CATION

P	0.000000000	0.000000000	1.103068000
C	0.000000000	0.000000000	-0.661516000
C	0.000000000	0.000000000	-3.443633000
C	1.222935000	0.000000000	-1.358746000
C	1.223189000	0.000000000	-2.755763000
C	2.368794000	0.000000000	-0.462960000
C	3.051909000	0.000000000	1.861404000
C	-1.222935000	0.000000000	-1.358746000
C	-1.223189000	0.000000000	-2.755763000
C	-2.368794000	0.000000000	-0.462960000
C	-3.051909000	0.000000000	1.861404000
H	0.000000000	0.000000000	-4.530265000
H	2.159369000	0.000000000	-3.308663000
H	2.533800000	0.000000000	2.823665000
H	3.400457000	0.000000000	-0.822578000
H	3.685382000	0.892376000	1.795818000
H	3.685382000	-0.892376000	1.795818000
H	-2.159369000	0.000000000	-3.308663000
H	-2.533800000	0.000000000	2.823665000
H	-3.400457000	0.000000000	-0.822578000

H	-3.685382000	0.892376000	1.795818000
H	-3.685382000	-0.892376000	1.795818000
N	2.065403000	0.000000000	0.790059000
N	-2.065403000	0.000000000	0.790059000

B3LYP, GROUND-STATE CATION

P	0.000000000	0.000000000	1.115941000
C	0.000000000	0.000000000	-0.650672000
C	0.000000000	0.000000000	-3.423321000
C	1.215158000	0.000000000	-1.346628000
C	1.215988000	0.000000000	-2.737086000
C	2.366370000	0.000000000	-0.460195000
C	3.087617000	0.000000000	1.844020000
C	-1.215158000	0.000000000	-1.346628000
C	-1.215988000	0.000000000	-2.737086000
C	-2.366370000	0.000000000	-0.460195000
C	-3.087617000	0.000000000	1.844020000
H	0.000000000	0.000000000	-4.504065000
H	2.147398000	0.000000000	-3.287005000
H	2.587903000	0.000000000	2.808989000
H	3.388868000	0.000000000	-0.832257000
H	3.715249000	0.888153000	1.772835000
H	3.715249000	-0.888153000	1.772835000
H	-2.147398000	0.000000000	-3.287005000
H	-2.587903000	0.000000000	2.808989000
H	-3.388868000	0.000000000	-0.832257000
H	-3.715249000	0.888153000	1.772835000
H	-3.715249000	-0.888153000	1.772835000
N	2.080825000	0.000000000	0.787430000
N	-2.080825000	0.000000000	0.787430000

27.AsAr, C_{2v}

OLYP, NEUTRAL

As	0.000000000	0.000000000	1.269268000
C	0.000000000	0.000000000	-0.591431000
C	0.000000000	0.000000000	-3.400271000
C	1.218066000	0.000000000	-2.706364000
C	1.231019000	0.000000000	-1.305696000
C	2.388334000	0.000000000	-0.461709000
C	3.222147000	0.000000000	1.794534000
C	-1.218066000	0.000000000	-2.706364000
C	-1.231019000	0.000000000	-1.305696000
C	-2.388334000	0.000000000	-0.461709000
C	-3.222147000	0.000000000	1.794534000
H	0.000000000	0.000000000	-4.486966000
H	2.157878000	0.000000000	-3.256301000
H	2.797333000	0.000000000	2.803499000
H	3.405692000	0.000000000	-0.862157000
H	3.856153000	0.891352000	1.684398000
H	3.856153000	-0.891352000	1.684398000

H	-2.157878000	0.000000000	-3.256301000
H	-2.797333000	0.000000000	2.803499000
H	-3.405692000	0.000000000	-0.862157000
H	-3.856153000	0.891352000	1.684398000
H	-3.856153000	-0.891352000	1.684398000
N	2.144326000	0.000000000	0.821978000
N	-2.144326000	0.000000000	0.821978000

B3LYP, NEUTRAL

As	0.000000000	0.000000000	1.285923000
C	0.000000000	0.000000000	-0.577311000
C	0.000000000	0.000000000	-3.376726000
C	1.209479000	0.000000000	-2.683713000
C	1.221650000	0.000000000	-1.290473000
C	2.381105000	0.000000000	-0.454642000
C	3.257039000	0.000000000	1.772658000
C	-1.209479000	0.000000000	-2.683713000
C	-1.221650000	0.000000000	-1.290473000
C	-2.381105000	0.000000000	-0.454642000
C	-3.257039000	0.000000000	1.772658000
H	0.000000000	0.000000000	-4.457707000
H	2.144502000	0.000000000	-3.231272000
H	2.855045000	0.000000000	2.783977000
H	3.388592000	0.000000000	-0.869365000
H	3.884394000	0.886720000	1.653092000
H	3.884394000	-0.886720000	1.653092000
H	-2.144502000	0.000000000	-3.231272000
H	-2.855045000	0.000000000	2.783977000
H	-3.388592000	0.000000000	-0.869365000
H	-3.884394000	0.886720000	1.653092000
H	-3.884394000	-0.886720000	1.653092000
N	2.159817000	0.000000000	0.821438000
N	-2.159817000	0.000000000	0.821438000

OLYP, GROUND-STATE CATION

As	0.000000000	0.000000000	1.266930000
C	0.000000000	0.000000000	-0.623414000
C	0.000000000	0.000000000	-3.401105000
C	1.221284000	0.000000000	-2.712289000
C	1.223242000	0.000000000	-1.311683000
C	2.396599000	0.000000000	-0.448153000
C	3.252428000	0.000000000	1.806253000
C	-1.221284000	0.000000000	-2.712289000
C	-1.223242000	0.000000000	-1.311683000
C	-2.396599000	0.000000000	-0.448153000
C	-3.252428000	0.000000000	1.806253000
H	0.000000000	0.000000000	-4.487434000
H	2.158745000	0.000000000	-3.263106000
H	2.823338000	0.000000000	2.811031000
H	3.407983000	0.000000000	-0.864777000
H	3.879114000	0.892237000	1.688236000

H	3.879114000	-0.892237000	1.688236000
H	-2.158745000	0.000000000	-3.263106000
H	-2.823338000	0.000000000	2.811031000
H	-3.407983000	0.000000000	-0.864777000
H	-3.879114000	0.892237000	1.688236000
H	-3.879114000	-0.892237000	1.688236000
N	2.178507000	0.000000000	0.820642000
N	-2.178507000	0.000000000	0.820642000

B3LYP, GROUND-STATE CATION

As	0.000000000	0.000000000	1.284070000
C	0.000000000	0.000000000	-0.610284000
C	0.000000000	0.000000000	-3.377432000
C	1.213510000	0.000000000	-2.690528000
C	1.214928000	0.000000000	-1.297564000
C	2.393321000	0.000000000	-0.443012000
C	3.289422000	0.000000000	1.784983000
C	-1.213510000	0.000000000	-2.690528000
C	-1.214928000	0.000000000	-1.297564000
C	-2.393321000	0.000000000	-0.443012000
C	-3.289422000	0.000000000	1.784983000
H	0.000000000	0.000000000	-4.458092000
H	2.145856000	0.000000000	-3.239810000
H	2.882772000	0.000000000	2.792663000
H	3.395019000	0.000000000	-0.870684000
H	3.909169000	0.888202000	1.658531000
H	3.909169000	-0.888202000	1.658531000
H	-2.145856000	0.000000000	-3.239810000
H	-2.882772000	0.000000000	2.792663000
H	-3.395019000	0.000000000	-0.870684000
H	-3.909169000	0.888202000	1.658531000
H	-3.909169000	-0.888202000	1.658531000
N	2.192515000	0.000000000	0.819639000
N	-2.192515000	0.000000000	0.819639000

28.SbAr, C_{2v}

OLYP, NEUTRAL

Sb	0.000000000	0.000000000	1.526851000
C	0.000000000	0.000000000	-0.532788000
C	0.000000000	0.000000000	-3.345102000
C	1.215917000	0.000000000	-2.649550000
C	1.227908000	0.000000000	-1.246567000
C	2.416787000	0.000000000	-0.442053000
C	3.442277000	0.000000000	1.717743000
C	-1.215917000	0.000000000	-2.649550000
C	-1.227908000	0.000000000	-1.246567000
C	-2.416787000	0.000000000	-0.442053000
C	-3.442277000	0.000000000	1.717743000
H	0.000000000	0.000000000	-4.432116000
H	2.156875000	0.000000000	-3.198126000

H	3.128789000	0.000000000	2.766058000
H	3.409112000	0.000000000	-0.903563000
H	4.061219000	0.891113000	1.541391000
H	4.061219000	-0.891113000	1.541391000
H	-2.156875000	0.000000000	-3.198126000
H	-3.128789000	0.000000000	2.766058000
H	-3.409112000	0.000000000	-0.903563000
H	-4.061219000	0.891113000	1.541391000
H	-4.061219000	-0.891113000	1.541391000
N	2.268065000	0.000000000	0.856732000
N	-2.268065000	0.000000000	0.856732000

B3LYP, NEUTRAL

Sb	0.000000000	0.000000000	1.529310000
C	0.000000000	0.000000000	-0.533669000
C	0.000000000	0.000000000	-3.331585000
C	1.208762000	0.000000000	-2.638464000
C	1.219805000	0.000000000	-1.242750000
C	2.407440000	0.000000000	-0.437774000
C	3.447417000	0.000000000	1.707644000
C	-1.208762000	0.000000000	-2.638464000
C	-1.219805000	0.000000000	-1.242750000
C	-2.407440000	0.000000000	-0.437774000
C	-3.447417000	0.000000000	1.707644000
H	0.000000000	0.000000000	-4.412604000
H	2.144008000	0.000000000	-3.185793000
H	3.136970000	0.000000000	2.750248000
H	3.393428000	0.000000000	-0.905444000
H	4.061810000	0.886662000	1.534057000
H	4.061810000	-0.886662000	1.534057000
H	-2.144008000	0.000000000	-3.185793000
H	-3.136970000	0.000000000	2.750248000
H	-3.393428000	0.000000000	-0.905444000
H	-4.061810000	0.886662000	1.534057000
H	-4.061810000	-0.886662000	1.534057000
N	2.270660000	0.000000000	0.850373000
N	-2.270660000	0.000000000	0.850373000

OLYP, GROUND-STATE CATION

Sb	0.000000000	0.000000000	1.541545000
C	0.000000000	0.000000000	-0.554516000
C	0.000000000	0.000000000	-3.341085000
C	1.216308000	0.000000000	-2.649994000
C	1.217868000	0.000000000	-1.246168000
C	2.422359000	0.000000000	-0.426517000
C	3.482297000	0.000000000	1.720751000
C	-1.216308000	0.000000000	-2.649994000
C	-1.217868000	0.000000000	-1.246168000
C	-2.422359000	0.000000000	-0.426517000
C	-3.482297000	0.000000000	1.720751000
H	0.000000000	0.000000000	-4.427345000

H	2.155391000	0.000000000	-3.198979000
H	3.171992000	0.000000000	2.768058000
H	3.406505000	0.000000000	-0.906020000
H	4.091481000	0.892098000	1.532168000
H	4.091481000	-0.892098000	1.532168000
H	-2.155391000	0.000000000	-3.198979000
H	-3.171992000	0.000000000	2.768058000
H	-3.406505000	0.000000000	-0.906020000
H	-4.091481000	0.892098000	1.532168000
H	-4.091481000	-0.892098000	1.532168000
N	2.301664000	0.000000000	0.857116000
N	-2.301664000	0.000000000	0.857116000

B3LYP, GROUND-STATE CATION

Sb	0.000000000	0.000000000	1.534468000
C	0.000000000	0.000000000	-0.563478000
C	0.000000000	0.000000000	-3.332803000
C	1.210559000	0.000000000	-2.645134000
C	1.211611000	0.000000000	-1.248743000
C	2.413212000	0.000000000	-0.425060000
C	3.474244000	0.000000000	1.715795000
C	-1.210559000	0.000000000	-2.645134000
C	-1.211611000	0.000000000	-1.248743000
C	-2.413212000	0.000000000	-0.425060000
C	-3.474244000	0.000000000	1.715795000
H	0.000000000	0.000000000	-4.413364000
H	2.143902000	0.000000000	-3.193258000
H	3.162383000	0.000000000	2.756613000
H	3.393619000	0.000000000	-0.900992000
H	4.080968000	0.887724000	1.535210000
H	4.080968000	-0.887724000	1.535210000
H	-2.143902000	0.000000000	-3.193258000
H	-3.162383000	0.000000000	2.756613000
H	-3.393619000	0.000000000	-0.900992000
H	-4.080968000	0.887724000	1.535210000
H	-4.080968000	-0.887724000	1.535210000
N	2.293684000	0.000000000	0.849828000
N	-2.293684000	0.000000000	0.849828000

29.BiAr, C_{2v}

OLYP, NEUTRAL

Bi	0.000000000	0.000000000	1.660377000
C	0.000000000	0.000000000	-0.492046000
C	0.000000000	0.000000000	-3.306244000
C	1.214166000	0.000000000	-2.609649000
C	1.227476000	0.000000000	-1.206109000
C	2.436630000	0.000000000	-0.427167000
C	3.568265000	0.000000000	1.667044000
C	-1.214166000	0.000000000	-2.609649000
C	-1.227476000	0.000000000	-1.206109000

C	-2.436630000	0.000000000	-0.427167000
C	-3.568265000	0.000000000	1.667044000
H	0.000000000	0.000000000	-4.393224000
H	2.155698000	0.000000000	-3.157770000
H	3.318812000	0.000000000	2.732772000
H	3.410622000	0.000000000	-0.928530000
H	4.177335000	0.891009000	1.456064000
H	4.177335000	-0.891009000	1.456064000
H	-2.155698000	0.000000000	-3.157770000
H	-3.318812000	0.000000000	2.732772000
H	-3.410622000	0.000000000	-0.928530000
H	-4.177335000	0.891009000	1.456064000
H	-4.177335000	-0.891009000	1.456064000
N	2.345891000	0.000000000	0.874731000
N	-2.345891000	0.000000000	0.874731000

B3LYP, NEUTRAL

Bi	0.000000000	0.000000000	1.662923000
C	0.000000000	0.000000000	-0.492337000
C	0.000000000	0.000000000	-3.291289000
C	1.207093000	0.000000000	-2.597057000
C	1.219055000	0.000000000	-1.200512000
C	2.426621000	0.000000000	-0.420907000
C	3.575437000	0.000000000	1.654727000
C	-1.207093000	0.000000000	-2.597057000
C	-1.219055000	0.000000000	-1.200512000
C	-2.426621000	0.000000000	-0.420907000
C	-3.575437000	0.000000000	1.654727000
H	0.000000000	0.000000000	-4.372556000
H	2.143233000	0.000000000	-3.143825000
H	3.332963000	0.000000000	2.715552000
H	3.393468000	0.000000000	-0.927457000
H	4.179357000	0.886609000	1.444185000
H	4.179357000	-0.886609000	1.444185000
H	-2.143233000	0.000000000	-3.143825000
H	-3.332963000	0.000000000	2.715552000
H	-3.393468000	0.000000000	-0.927457000
H	-4.179357000	0.886609000	1.444185000
H	-4.179357000	-0.886609000	1.444185000
N	2.348430000	0.000000000	0.869619000
N	-2.348430000	0.000000000	0.869619000

OLYP, GROUND-STATE CATION

Bi	0.000000000	0.000000000	1.674363000
C	0.000000000	0.000000000	-0.519792000
C	0.000000000	0.000000000	-3.308183000
C	1.214424000	0.000000000	-2.616059000
C	1.217672000	0.000000000	-1.210605000
C	2.438586000	0.000000000	-0.412717000
C	3.594625000	0.000000000	1.674477000
C	-1.214424000	0.000000000	-2.616059000

C	-1.217672000	0.000000000	-1.210605000
C	-2.438586000	0.000000000	-0.412717000
C	-3.594625000	0.000000000	1.674477000
H	0.000000000	0.000000000	-4.394614000
H	2.153918000	0.000000000	-3.164658000
H	3.342538000	0.000000000	2.737429000
H	3.406898000	0.000000000	-0.925048000
H	4.193986000	0.891901000	1.454656000
H	4.193986000	-0.891901000	1.454656000
H	-2.153918000	0.000000000	-3.164658000
H	-3.342538000	0.000000000	2.737429000
H	-3.406898000	0.000000000	-0.925048000
H	-4.193986000	0.891901000	1.454656000
H	-4.193986000	-0.891901000	1.454656000
N	2.368511000	0.000000000	0.873861000
N	-2.368511000	0.000000000	0.873861000

B3LYP, GROUND-STATE CATION

Bi	0.000000000	0.000000000	1.662542000
C	0.000000000	0.000000000	-0.528316000
C	0.000000000	0.000000000	-3.298752000
C	1.209136000	0.000000000	-2.609870000
C	1.211912000	0.000000000	-1.211682000
C	2.431487000	0.000000000	-0.411529000
C	3.590298000	0.000000000	1.669753000
C	-1.209136000	0.000000000	-2.609870000
C	-1.211912000	0.000000000	-1.211682000
C	-2.431487000	0.000000000	-0.411529000
C	-3.590298000	0.000000000	1.669753000
H	0.000000000	0.000000000	-4.379345000
H	2.142809000	0.000000000	-3.157746000
H	3.336071000	0.000000000	2.726201000
H	3.394925000	0.000000000	-0.923387000
H	4.187286000	0.887644000	1.457889000
H	4.187286000	-0.887644000	1.457889000
H	-2.142809000	0.000000000	-3.157746000
H	-3.336071000	0.000000000	2.726201000
H	-3.394925000	0.000000000	-0.923387000
H	-4.187286000	0.887644000	1.457889000
H	-4.187286000	-0.887644000	1.457889000
N	2.364642000	0.000000000	0.866299000
N	-2.364642000	0.000000000	0.866299000
